# Comparison of morphological, DNA barcoding, and metabarcoding characterizations of freshwater nematode communities

**DOI:** 10.1002/ece3.6104

**Published:** 2020-02-15

**Authors:** Janina Schenk, Nils Kleinbölting, Walter Traunspurger

**Affiliations:** ^1^ Department of Animal Ecology Bielefeld University Bielefeld Germany; ^2^ Center for Biotechnology Bielefeld University Bielefeld Germany

**Keywords:** diversity, metazoan, molecular species identification, taxonomic assignment

## Abstract

Biomonitoring approaches and investigations of many ecological questions require assessments of the biodiversity of a given habitat. Small organisms, ranging from protozoans to metazoans, are of great ecological importance and comprise a major share of the planet's biodiversity but they are extremely difficult to identify, due to their minute body sizes and indistinct structures. Thus, most biodiversity studies that include small organisms draw on several methods for species delimitation, ranging from traditional microscopy to molecular techniques. In this study, we compared the efficiency of these methods by analyzing a community of nematodes. Specifically, we evaluated the performances of traditional morphological identification, single‐specimen barcoding (Sanger sequencing), and metabarcoding in the identification of 1500 nematodes from sediment samples. The molecular approaches were based on the analysis of the 28S ribosomal large and 18S small subunits (LSU and SSU). The morphological analysis resulted in the determination of 22 nematode species. Barcoding identified a comparable number of operational taxonomic units (OTUs) based on 28S rDNA (*n* = 20) and fewer OTUs based on 18S rDNA (*n* = 12). Metabarcoding identified a higher OTU number but fewer amplicon sequence variants (AVSs) (*n* = 48 OTUs, *n* = 17 ASVs for 28S rDNA, and *n* = 31 OTUs, *n* = 6 ASVs for 18S rDNA). Between the three approaches (morphology, barcoding, and metabarcoding), only three species (13.6%) were shared. This lack of taxonomic resolution hinders reliable community identifications to the species level. Further database curation will ensure the effective use of molecular species identification.

## Graphical abstract

A comparison of the accuracy of molecular‐based species identification (DNA barcoding and metabarcoding) compared to microscopic identification exemplarily carried out on nematodes. The dominant species could be recovered by all methods, while less abundant were better recovered by microscopic analysis.

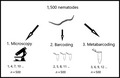

## 1 INTRODUCTION

Accurate species identifications are important for biomonitoring, and many environmental indices rely on identification to the genus or species level. Small organisms, such as nematodes, contribute substantially to the planet's biodiversity. However, their identification is particularly challenging and often possible only by taxonomic experts (Geiger et al., 2016). Nematodes are estimated to exceed one million species (Blaxter et al., 2005; Lambshead, 1993) and account for numerous critical functions in the ecosystem, ranging from nutrient production and nutrient cycling to key links in food webs and catalysts in decomposition processes (Majdi & Traunspurger, 2015; Schmid‐Araya et al., 2002; Traunspurger, Bergtold, & Goedkoop, 1997; Weber & Traunspurger, 2015). Previous studies have recognized the importance of this organismal group and investigated nematode diversity, but the characterizations were restricted to the genus level, as identification problems limited species‐level determinations (Fontaneto, Flot, & Tang, 2015; Hebert, Ratnasingham, & deWaard, 2003).

With the decline in taxonomic expertise, newly developed molecular methods are being increasingly used in species identification, especially of organisms at the microscopic and microbial scales (Creer et al., 2016). Other applications of molecular methods include whole‐genome analyses, the determination of evolutionary patterns in phylogeographic and phylogenetic studies (Blaxter et al., 2005; Derycke et al., 2008; Holterman et al., 2006; Junqueira et al., 2016), and initiatives aimed at collecting the planet's biodiversity in molecular databases. The latter relies on species identification using a single short gene fragment, the so‐called barcoding approach (Hebert et al., 2003; Stoeckle & Stoeckle, 2003). The short fragments are usually from gene regions that evolve fast enough to enable differentiations of closely related species, but are also conservative enough to allow universal primer design (Floyd, Abebe, Papert, & Blaxter, 2002). For example, the large ribosomal subunit (LSU) evolves conservatively but still accumulates differences between closely related species (Markmann & Tautz, 2005). Its utility in distinguishing between nematode species has been demonstrated in several studies (Geiger et al., 2016; Ristau, Steinfartz, & Traunspurger, 2013; Schenk, Hohberg, Helder, Ristau, & Traunspurger, 2017). The small ribosomal subunit (SSU) evolves more conservatively and is therefore often used to distinguish between species that are not closely related (Armenteros et al., 2014; Nassonova, Smirnov, Fahrni, & Pawlowski, 2010; Prosser, Velarde‐Aguilar, Leon‐Regagnon, & Hebert, 2013). The use of the 18S gene in molecular studies is currently well‐accepted in NGS studies (Chariton et al., 2014; Fonseca et al., 2010) and phylogenetic analyses (Holterman et al., 2006; van Megen et al., 2009). The cytochrome c oxidase I (COI) gene fragment was initially considered as the marker of choice for barcoding purposes (Hebert et al., 2003) due to its ubiquity, as the COI gene is present in all cells, as well as its high interspecific and low intraspecific genetic variation (Derycke, Vanaverbeke, Rigaux, Backeljau, & Moens, 2010). However, while the COI gene is suitable for many organisms, its performance in several animal groups is poor because of the low amplification success resulting from mutations in primer‐binding regions (Blaxter et al., 2005; De Ley et al., 2005).

Barcoding studies based on the use of short gene fragments for metazoa have investigated tardigrades, rotifers, mites, collembolans, and nematodes (Ball, Hebert, Burian, & Webb, 2005; Blaxter, Elsworth, & Daub, 2004; Fontaneto et al., 2015). The emergence of NGS has revolutionized the field of molecular taxonomy. Among its applications are the sequencing of short gene fragments, so‐called amplicons (metabarcoding), mitogenomic analyses, and the generation of whole genomes (Ji et al., 2013; Junqueira et al., 2016; Tang et al., 2015). NGS, and specifically metabarcoding, allows biodiversity to be captured at an unprecedented level of detail. Furthermore, the bulk DNA extraction method used in NGS can simplify the analysis of complete communities (Elbrecht & Leese, 2017). However, although NGS amplicon sequencing has been in use for more than a decade (Porazinska et al., 2009), its methods have yet to be standardized, including for nematode communities. Additional shortcomings currently include a lack of a reliable quantification methods and the problem of PCR bias, which can result in an over‐ or under‐amplification of the DNA of certain species (Geisen, Laros, Vizcaíno, Bonkowski, & Groot, 2015; Kebschull & Zador, 2015; Tang et al., 2015), as well as incomplete reference databases (Abad et al., 2016; Holovachov, 2016).

Metabarcoding has facilitated studies of small multicellular organisms, either whole communities or specific groups, with marine eukaryotes being a frequent focus (Brannock & Halanych, 2015; Dell'Anno, Carugati, Corinaldesi, Riccioni, & Danovaro, 2015; Haenel, Holovachov, Jondelius, Sundberg, & Bourlat, 2017). It can also be used in combination with morphological analyses, as demonstrated in studies of estuarine plankton (Abad et al., 2016; Harvey, Johnson, Fisher, Peterson, & Vrijenhoek, 2017; Leasi et al., 2018) and nematodes (Holovachov, 2016; Macheriotou et al., 2019) in marine habitats but also diatoms and other small organisms in freshwater habitats (Keck, Vasselon, Rimet, Bouchez, & Kahlert, 2018; Rimet, Vasselon, A.‐Keszte, & Bouchez, 2018). For nematodes in soil and marine habitats, however, combined microscopy and metabarcoding investigations have been carried out only at the family level (Darby, Todd, & Herman, 2013; Griffiths, Groot, Laros, Stone, & Geisen, 2018; Holovachov, Haenel, Bourlat, & Jondelius, 2017; Treonis et al., 2018), and direct comparisons of the performances of morphological identification, barcoding, and metabarcoding at the species level are still scarce (Leasi et al., 2018).

Therefore, in this study, we compared the results of a morphological analysis with those from single‐specimen barcoding and metabarcoding to determine the most accurate approach to specimen identification. We predicted that the three methods would identify a similar number of species and certainly all dominant species contributing to the community. In addition, we expected that the proportion of species identified morphologically and by single‐specimen barcoding would be comparable, whereas species abundance estimates obtained with metabarcoding would differ due to PCR and sequencing biases.

## 2 MATERIAL AND METHODS

Samples were collected at the extensively studied Furlbach stream (51.895392°N, 8.715517°E; Traunspurger, Threis, & Majdi, 2015) in May 2017. Sediment samples (~4 m^2^) were decanted directly in the field by skimming the upper 10 cm of sediment, transferring it into a bucket and stirring it for 30 s. After the suspension was left to stand for 15 s, the supernatant was poured over a 10‐µm sieve and the contents of the sieve were transferred to a sampling bottle. This procedure was repeated three times. The sample was stored at 4°C until used in nematode isolation.

**Figure 1:**
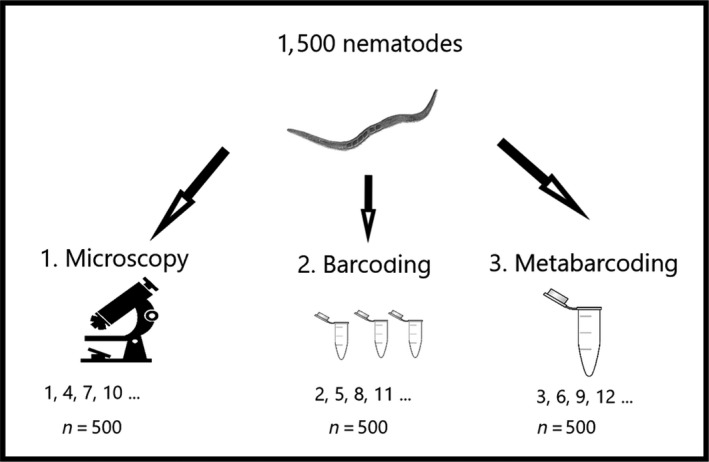
Overview of nematode sorting for the three approaches to species identification

Living nematodes were selected under stereomicroscopic guidance (40 × magnification). From the 1,500 nematodes in the sediment sample, 500 were randomly selected and assigned to each of the three treatments to generate comparable subsets (Figure 1). The 500 nematodes to be identified morphologically were fixed following the method of Seinhorst (1962) and prepared on permanent glycerin slides. In many diversity analyses (Hodda & Abebe, 2006; Rzeznik‐Orignac et al., 2017), 100 nematodes are identified; thus, our inclusion of 500 nematodes ensured that similar communities were achieved although only one subset would be analyzed morphologically. The 500 nematodes for single barcoding were individually transferred into small tubes containing 20 µl of lysis buffer (50 nM KCl, 10 mM Tris (pH 8.5), 2.5 mM MgCl_2_, 0.5% Triton X‐100, 0.5% Tween 20) and stored at − 20°C for at least 24 hr, while those for metabarcoding (NGS) were transferred as a whole community into the DNA extraction buffer provided with the NucleoSpin XS DNA extraction kit (Macherey and Nagel, Düren, Germany).

### 2.1 Morphological analyses

A Leica Dialux 20 microscope and a magnification of 400‐ to 1,200 × were used to identify nematode individuals to the species level. The morphological identification was conducted by Prof. Dr. Traunspurger, mainly following the method of Andrássy (2005, 2007, 2009 and reference therein). A detailed list of the species inventory, including life stage and sex, is given in the (Table S1).

### 2.2 Barcoding

Prior to sequencing, a reference dataset for taxonomic assignment was compiled from an NCBI search to retrieve sequences corresponding to the species identified in the morphological analysis as well as typical freshwater nematode sequences (Table S2). This avoided uninformative assignments such as “uncultured eukaryote” and “Nematoda environmental sample.” In addition, for each species detected in our study, the number of deposited sequences was noted in order to assess the current status of reference database curation.

Single nematodes frozen in barcoding lysis buffer were thawed, and 1.5 µl of proteinase K (20 mg/ml) was added to each sample, followed by lysis for 70 min at 65°C and 10 min at 95°C. The gene fragments were then amplified using the primer pair 1274/706 (5’–GACCCGTCTTGAAACACGGA‐3’/5’‐GCCAGTTCTGCTTACC‐3‘) designed by Markmann & Tautz (2005) for the D3–D5 region of the LSU (28S gene region hereafter) and the primer pair F04/R22 (5′‐GCTTGTCTCAAAGATTAAGCC‐3′/5′‐GCCTGCTGCCTTCCTTGGA‐3′) designed for the V1–V2 region of the SSU (18S gene region hereafter; Fonseca et al., 2010). PCRs for the 28S gene region were carried out in 20‐µl volumes consisting of 2 µl of genomic DNA, 14.2 µl of ultrapure water, 0.6 µl of each primer (10 µM), 2 µl of reaction buffer Y (Peqlab, Erlangen), 1 µl of dNTPs (Roth, Karlsruhe), and 0.1 µl of Taq‐DNA‐polymerase (Peqlab). PCRs for the other gene region differed by the addition of 2 µl of MgCl_2_ to each reaction and the use of only 1.5 µl of genomic DNA. PCR conditions were 94°C for 2.5 min, followed by 30 cycles of 30 s at 95°C, 30 s at 55°C, and 60 s at 72°C, a final extension at 72°C for 7 min and cooling at 6°C.

Amplification results were checked by electrophoresis on an agarose gel (2%) and ethidium bromide staining. If the amplification was successful, evidenced by a positive band in the gel, the reaction product was cleaned using ExoSap exonuclease I (20 U/µl; Thermo Fisher Scientific, Waltham, MA) and shrimp alkaline phosphatase (1 U/µl; Affymetrix, Santa Clara, CA) in an 18‐min incubation at 37°C and a 15‐min incubation at 80°C. Sequencing was carried out on ABI PRISM 377, 3,100, and 3,700 sequencers (Applied Biosystems, Weiterstadt, Germany) at the CeBiTec Bielefeld, using BigDye Terminator v3.1 chemistry and the same primer pairs as used for sequencing.

Forward and reverse sequences were merged into contigs with an overlap of at least 20 bp using ChromasPro (Technelysium Pty Ltd, South Brisbane, Australia) and manually checked for ambiguous bases. All sequences of sufficient quality (no N‐characters) were further processed with the RDP classifier (Wang, Garrity, Tiedje, & Cole, 2007). The RDP classifier was trained using the curated reference database (see above). Taxonomic assignments were made based on the lowest level that provided a confidence score of at least 80%. Only classifications at the genus or species level were considered; others were regarded as uncertain. The sequences were collapsed into haplotypes and sorted into OTUs using the R (R Core Team, 2013) package “splits” (http://splits.r-forge.r-project.org/) with the *gmyc* function. The Jukes–Cantor model was used for both genetic markers, as previously described, and defined by jModelTest (Posada, 2008). New sequences were deposited at the NCBI under the accession numbers MK379606‐MK379948 and MK382985‐MK383328. A detailed overview of the taxonomic assignments for the barcoded specimens is given in (Table S3).

The packages “iNEXT” (Hsieh, Ma, & Chao, 2016) and “fossil” (Vavrek, 2011) in R were used to calculate rarefaction curves with the *iNEXT* function and to estimate species richness with the *jack1* function, for both morphological and barcoding data (Figure S1).

### 2.3 Metabarcoding

DNA of the metabarcoding sample was extracted using the NucleoSpin tissue XS kit (Macherey‐Nagel, Düren, Germany) according to the manufacturer's protocol, but with a lysis time in a rocking water bath of 10 hr, rather than the suggested 1–3 hr. The PCR primers were identical to those used for barcoding (F04/R22 and 1274/706, see above). PCRs were carried out for 30 cycles using a mix of high‐fidelity and standard polymerases (MyTaq) and the following conditions: 1 min 96°C predenaturation; 96°C for 15 s, 58°C for 30 s, and 70°C for 90 s. Amplicons were quality checked, and an individual index adaptor was ligated in a second 10‐cycle PCR using the same conditions. The PCR products (20 ng each) were submitted for Illumina MiSeq (2 × 300 bp) sequencing with V3 chemistry at LGC Genomics (Berlin) in a shared run. One million raw reads of the sequencing run were delivered demultiplexed.

Bioinformatics downstream analyses followed two approaches, resulting in operational taxonomic units (OTUs) and amplicon sequence variants (ASVs). The OTU analyses were performed with mothur and largely followed the MiSeq standard operation procedure until taxonomic classification (Kozich, Westcott, Baxter, Highlander, & Schloss, 2013; Schloss et al., 2009). Demultiplexed reads were combined using mothurs make.contigs with default parameters. Over 72% of the reads could be merged, showing that the overlap was sufficient. The average Phred score of the forward reads was >30 even toward the end of the read, while reverse reads had an average score >20 toward the ends. Only reads containing both primer sequences were retained in the dataset. Cutadapt (Martin, 2011) was used to remove primer sequences from the combined reads with a default error rate of 0.1. In a further filtering step, reads with ambiguous bases, homopolymers >10 bases, and unexpectedly short or long reads (allowed ranges: 333–367 for 18S, and 471–516 for 28S, lower and upper limits were selected as the 2.5% and 97.5% percentile, respectively) were excluded as well using screen_seqs (default parameters). The resulting sequences were aligned using the SILVA 132 reference alignment (Martin, 2011) to determine the spanned 18S or 28S rDNA region within the alignment. Sequences not spanning this region were eliminated and overhangs were cut. The sequences were then clustered into OTUs (pre.cluster with single‐linkage) with a maximum difference of 4 (18S) or 5 (28S), equivalent to a ~99% clustering threshold. The resulting OTUs were then checked for chimeras, which were removed using UCHIME (Edgar, Haas, Clemente, Quince, & Knight, 2011). The ASVs were generated using the dada2 pipeline, as described by Callahan et al. (2016). Adapter sequences were removed with cutadapt, as described above, and a custom dada2 script in R was used as reported in detail at: https://benjjneb.github.io/dada2/tutorial.html. The parameters were slightly changed (length cutoff (280,250), maxEE = c (2,2), truncQ = 2).

The RDP classifier was used to further annotate the OTUs and ASVs (using the same model as for the taxonomic classification of the sanger sequences). Identical classifications at the species and genus level were combined into new OTUs.

The placement of the OTUS and ASVs was checked by building phylogenetic trees with MEGA (Kumar, Stecher, & Tamura, 2016) based on maximum likelihood (500 generations, Jukes‐Cantor model as calculated by jModelTest) and using all newly generated sequences in this study as well as the reference dataset.

### 2.4 Comparisons of the methods

Venn diagrams were used to visualize the concordance between morphological, barcoding, and metabarcoding identifications at the species level. A genus‐level comparison was also conducted, as several of the OTUs and ASVs could not be assigned at higher taxonomic levels (Figure S2).

## 3 RESULTS

### 3.1 Morphological analysis

In the morphological analysis, 483 specimens could be assigned taxonomically to the species level and 485 to the genus level. Overall, 22 nematode species and one Mermithidae species were identified. Among the 22 nematode species, 7 were dominant (>2% abundance) while the others were detected only in lower abundances (<2%). Seven species were single finds (0.21% abundance). The most abundant species was *Theristus agilis* (37.32%), followed by *Anaplectus grandepapillatus* (18.56%), *Chromadorita leuckarti* (10.10%), *Tripyla setifera* (9.48%), *Semitobrilus pellucidus *(9.07%), *Theristus vesentinae* (3.30%), and *Eumonhystera vulgaris* (2.27%). An overview of all nematode species found is given in Table 1. The 22 discovered species were 77.4% of the estimated species richness (jackknife estimator), and the rarefaction curve showed an estimated increase of 26.1% (6 species) within the next 500 specimens (Figure S1).

**Table 1:** List of species identified for morphology, barcoding, and metabarcoding of 18S and 28S rDNA, as is whether 28S, 18S, or COI sequences were found in the ncbi database, as well as the according number of deposited sequences. The abundance (%) of the total nematodes identified morphologically is given (*n* = 485), OTU assignments for the 28S and 18S gene fragments are shown as well, together with the annotation via RDP classifier. The proportion of the OTUs for 28S and 18S barcoding is based on the total number of successfully amplified sequences (*n* = 344 for 28S and *n* = 343 for 18S). For metabarcoding, OTU and ASV percentage is based on the number of reads remaining after bioinformatics pipeline. Classifications below the genus level are summoned due to overview reasons, if the same classification was reached; the number of OTUs/ASVs for each marker in given in parentheses

Species	ncbi	Nb of sequences	Microscopy	Barcoding_28S	NGS_28S		Barcoding_18S	NGS_18S	
			(%)	(%)	%	%	(%)	%	%
			*n* = 485	*n* = 344	OTUs	ASVs	*n* = 343	OTUs	ASVs
*Achromadora terricola* (de Man, 1880)	18S	1	1.24						
*Allodorylaimus _1*								0.03	
*Anaplectus grandepapillatus* (Ditlevsen, 1928)	18S	2	18.56						
*Anaplectus granulosus* (Bastian, 1865)	28S	4		18.6	3.42	1.1			
*Anaplectus porosus* Allen & Noffsinger, 1968	28S&18S	4					17.25	5.1	5.81
*Anaplectus_2*								0.02	
*Aphelenchoides* sp.	28S&18S&COI	>100	0.21						
*Chromadorita leuckarti* (de Man, 1876)	28S&18S	54	10.10	9.59	7.65	2.51	10.23	7.11	7.09
*Cylindrolaimus melancholicus* de Man, 1880	–	0	0.82						
*Cylindrolaimus* sp.	18S	3					0.29		
*Ethmolaimus pratensis* de Man, 1880	18S&28S	10	1.03					0.02	
*Epidorylaimus cf. agilis* (de Man, 1880)			0.21						
*Eudorylaimus* cf. *carteri* (Bastian, 1865)	28S&18S	9	0.21						
*Eudorylaimus* sp.	28S&18S	22							
*Eudorylaimus_28S_a*				0.29					
*Eumonhystera* cf. *barbata* Andrássy, 1981	–	0	0.21						
*Eumonhystera dispar* (Bastian, 1865)	–	0	0.82						
*Eumonhystera filiformis/hungarica*	28S&18S	12		0.29				0.21	
*Eumonhystera longicaudatula* (Gerlach & Riemann, 1973)	18S	1	1.86						
*Eumonhystera vulgaris* (de Man, 1880)	18S	1	2.27					0.04	
*Eumonyhstera pseudobulbosa* (Daday, 1896)	–	0	0.21						
*Eumonhystera_18S_a*							1.75	0.02	
*Eumonhystera_10*								0.04	
*Eumonhystera_3*								0.03	
*Filenchus* sp.	28S&18S	38	0.21						
*Hofmaenneria niddensis* Schneider, 1940	–	0	1.65						
*Ironus longicaudatus* de Man, 1884	28S&18S	4		0.29	0.13	0.08			
*Ironus_18S_a*							0.29	0.06	
Mermithidae			0.41						
*Monhystera paludicola* de Man, 1881	28S&18S	15	0.41						
*Monhystera stagnalis* Bastian, 1865	28S&18S	11	0.21						
*Monhystera sp.*	28S + 18S	32							
*Monhystera wangi* Wu & Hoeppli, 1929	28S	1			0.38				
*Monhystera_28S_a*				1.16	0.08				
*Monhystera_28S_b*				0.87					
*Monhystera_28S_c*				0.58		0.1			
*Monhystera_28S_d*				0.58					
*Monhystera_28S_e*				0.29					
*Monhystera_10*					0.08				
*Monhystera_2*						0.03			
*Monhystera_3*								0.06	
*Mononchus truncatus* Bastian, 1865	28S&18S	8		0.29			0.29		
*Mononchus_18S_a*							0.29		
*Plectus aquatilis* Andrássy, 1985	28S&18S&COI	17						0.09	
*Plectus_11*						0.83			
*Plectus_2*					2.83				
*Plectus_3*					1.69				
*Plectus_6*					0.08				
*Prismatolaimus dolichurus* De Man, 1880	28S&18S	10			0.08				
*Prismatolaimus intermedius* (Bütschli, 1873)	18S	3	0.21						
*Rhomborhabditis regina* (Schulte & Poinar, 1991)	28S&18S	9			0.08				
*Semitobrilus pellucidus* a (Bastian, 1865)	28S&18S	13	9.07	9.01	31.75	5.79			
*Theristus sp.*	28S&18S&COI	22					50.88	68.03	69.75
*Theristus agilis* (de Man, 1880)	18S	3	37.32					0.02	
*Theristus vesentinae* (Andrássy 1962)	–	0	3.30						
*Theristus_5*								0.02	
*Theristus_7*								0.02	
*Tridentulus sp.*	18S	2						0.07	
*Tripyla setifera* Bütschli, 1873	28S	15	9.48	4.36	4.82	1.08			
*Triypla* sp.	28S&18S	77							
Tripyla_18S_a							5.56	1.86	1.18
Sanger_18S_1 (Tobrilidae)							9.94	13.83	12.71
Sanger _28S_1 (Chromadorea)				50.29	10.87	0.75			
Sanger _28S_2 (Chromadorea)				2.03	2.37	0.95			
Sanger _18S_2 (Chromadorea)							2.92	0.06	
Sanger _18S_3 (Tobrilidae)							0.29		
Sanger _28S_3 (Chromadorea)				0.29					
Sanger _28S_4 (Triplonchida)				0.29					
Sanger _28S_5 (Nematoda)				0.29					
Sanger _28S_6 (Monhysteridae)				0.29					
Sanger _28S_7 (Nematoda)				0.29					
Chromadorea (OTU: 18S = 1,28S = 18; ASV: 28S = 4)					19.45	83.67		0.02	
Plectidae (OTU: 28S = 4; ASV: 28S = 2)					6.43	2.06			
Tobrilidae (OTU: 18S = 7,28S = 2; ASV: 18S = 1,28S = 1)					0.25			0.18	
Chromadorida (OTU: 18S: 1; ASV: 18S: 1)								3.04	3.45
Nematoda (OTU: 18S = 1,28S = 9)					7.53	1.03		0.03	

a 28S reference database gave results as *Tobrilus pellucidus* instead of *Semitobrilus pelludicus*, although the same species is meant.

### 3.2 Barcoding

From the 500 nematodes genetically analyzed using the 28S and 18S rDNA gene regions, a sequence for at least one of the markers was obtained for 380 (76%) individuals, including successful amplification of both markers in 308 (61.6%) individuals. No sequence was obtained for 120 (24%) of the nematodes.

**Figure A1:**
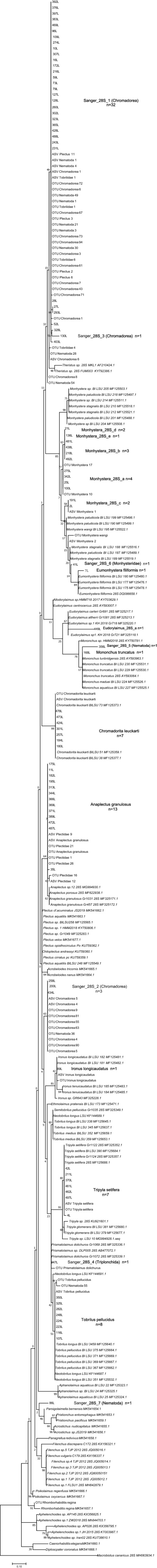
Maximum likelihood tree based on 500 generations for the 28S gene region performed using all newly generated Sanger sequences and the reference dataset (highlighted in italics). Sequences generated in this study are abbreviated with a number (1–500 L) and color‐coded according to the OTU they were sorted into based on *gmyc*. The taxonomic assignment by RDP is shown in parentheses if the resolution was lower than genus level

**Figure A2:**
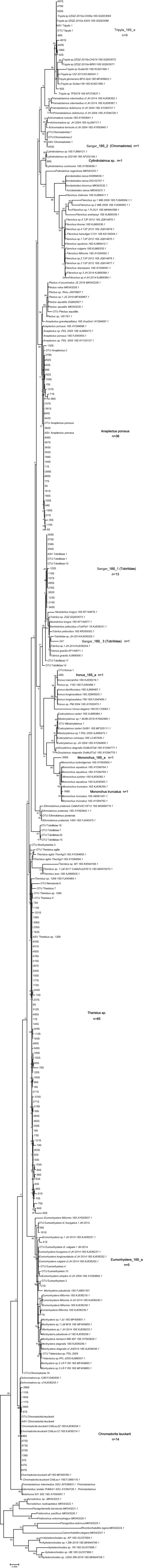
Maximum likelihood tree based on 500 generations for the 18S gene region, performed for all newly generated Sanger sequences and the reference dataset (highlighted in italics). Sequences generated in this study are abbreviated with a number (1–500 S) and color‐coded according to the OTU they were sorted into based on *gmyc*. The taxonomic assignment by RDP is shown in parentheses if the resolution was lower than genus level

Based on the 520‐bp 28S rDNA gene fragment, 20 OTUs (66.7% of the estimated species richness) were identified by *gmyc*. However, the most abundant OTU (Sanger_28S_1, 50.29% of all successfully amplified specimens) could not be annotated but was instead classified as Chromadorea and assigned phylogenetically close to *Theristus* (Figure A1). Other frequent OTUs were *Anaplectus granulosus* (18.60%), *Chromadorita leuckarti* (9.59%), *Tobrilus pellucidus^1^* (9.01%), *Tripyla setifera* (4.36%), Sanger _28S_2 (2.03%), which was classified as Chromadorea, and *Monhystera_28S_a* (1.16%). The abundances of the remaining OTUs were <1% (Table 1). Sequences ~400 bp in length and belonging to the V1–V2 region of the 18S rDNA gene fragment resulted in 12 OTUs (70.6% of the estimated species richness), with those of *Theristus* sp. (50.88%), *Anaplectus porosus* (17.25%), *Chromadorita leuckarti* (10.23%), Sanger_18S_1 (9.94%), *Triyla_18S_a* (5.56%), Sanger _18S_3 (2.92%), and *Eumonhystera_18S_a* (1.75%) occurring with the highest frequency. Sanger_18S_1 (Tobrilidae) was placed close to *Tobrilus* in the phylogenetic analysis (Figure A2). The frequency of the remaining OTUs was ≤ 1%. A list of all taxonomic assignments based on barcoding with both markers is given in Table 1.

### 3.3 Metabarcoding

After quality filtering and the removal of chimeric reads, the OTU dataset was reduced from 12,955 read pairs to 10,116 merged reads (for 18S) and from 12,847 read pairs to 2,365 merged reads (for 28S). An overview of the reduction of the read number during the bioinformatic pipeline process is given in the (Table S4). For the 28S marker, 48 OTUs were identified after RDP classifier annotation, although many could not be conclusively resolved at the taxonomic level and only class assignments were possible (Table 1). At the species and genus levels, the most frequent OTUs were *Tobrilus pellucidus* (31.75%), Sanger _28S_1 (10.87%; as found by Sanger sequencing)*, Chromadorita leuckarti* (7.65%), *Tripyla setifera* (4.82%), *Anaplectus granulosus* (3.42%), and *Plectus_2* (2.83%). The dada2 pipeline resulted in 6,006 reads that consisted of 17 ASVs. The most frequent was not classified (Chromadorea_1; 81.77%), followed by *T. pellucidus* (5.79%), *C. leuckarti* (2.51%), *A. granulosus* (1.1), and *T. setifera* (1.08%) (Table 1).

The OTU‐pipeline for the 18S gene fragment resulted in 31 OTUs classified by the RDP classifier. The highest number of reads belonged to *Theristus* sp. (68.03%), followed by Sanger _18S_1 (Tobrilidae) (13.83%), *C. leuckarti* (7.11%), *Anaplectus porosus* (5.10%), Chromadorida_1 (3.04%), and *Tripyla_1* (1.86%) (Table 1). For the dada2 pipeline, only 6 ASVs were recovered from 1,015 reads, with the most frequent belonging to *Theristus sp_1* (69.75%), followed by an unclassified OTU (Tobrilidae; 12.71%), *C. leuckarti* (7.09%), *A. porosus* (5.81%), Chromadorida (3.45%), and *Tripyla* (1.18%) (Table 1).

### 3.4 Comparisons between methods

**Figure 2:**
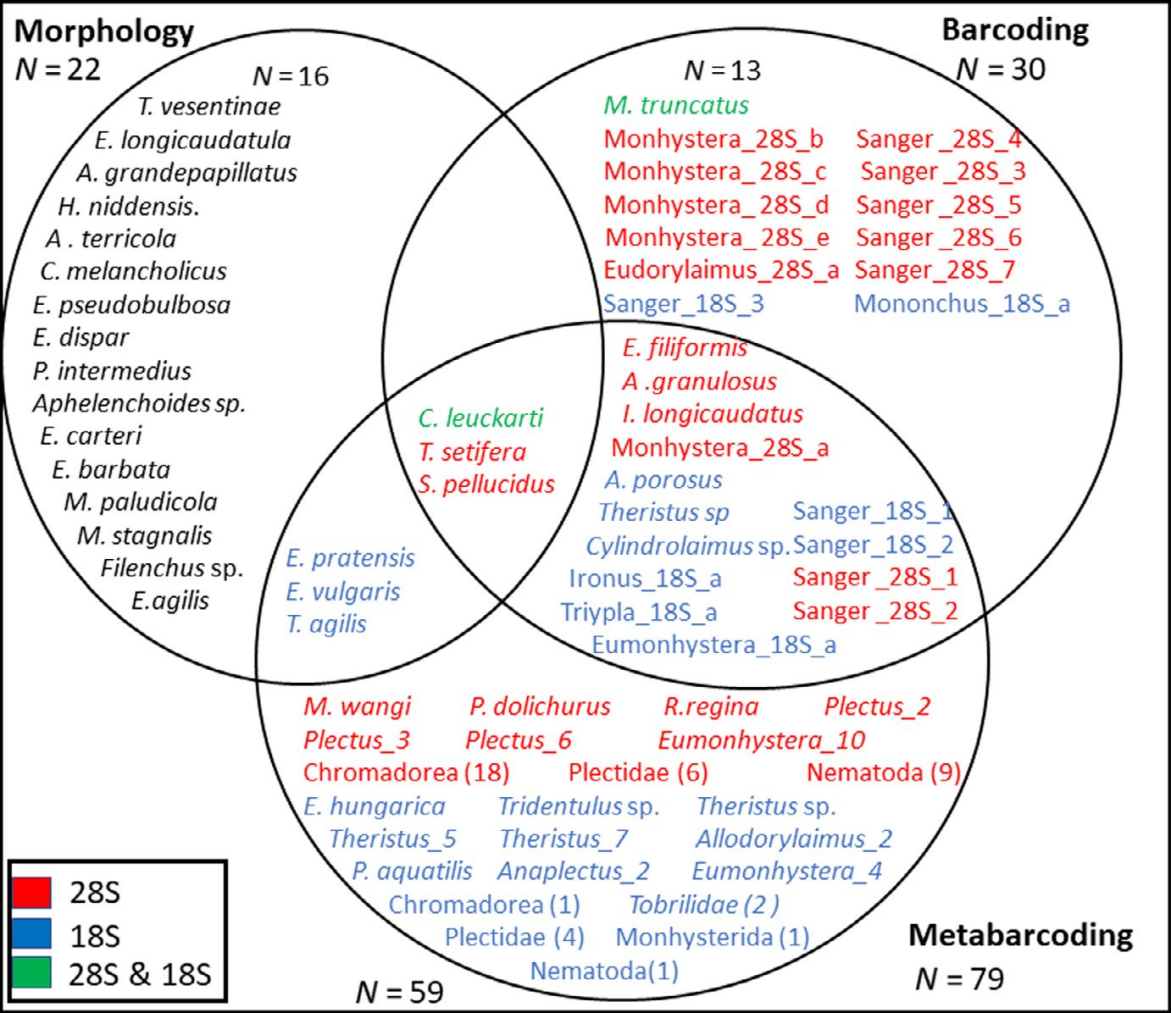
Venn diagrams showing the overlap in species identification by the three approaches. The number of species or genera found by each method is indicated outside the circles. Species identified by 18S rDNA and 28S rDNA OTUs are shown in color. OTUs classified below the genus level were combined. The total number of OTUs is shown in parentheses

From the morphologically identified species, six (27.3%) were recovered by one of the molecular approaches (Figure 2). Barcoding and metabarcoding recovered three species (13.6%), of which only one (4.5%) was detected by both genetic markers. From the 30 OTUs found by barcoding, 17 (56.7%) were also recovered by metabarcoding, while 17 (21.5%) of the metabarcoding OTUs (*n* = 79) were also found by barcoding. At the genus level, 62.5% of the morphologically identified genera were recovered by at least one molecular approach (Fig. S2b), indicating that taxonomic assignment is hampered by missing reference sequences.

## 4 DISCUSSION

This study compared three methods of species identification: morphological inspection and two molecular approaches, barcoding, including Sanger sequencing, and high‐throughput metabarcoding (NGS). Our results showed that the different methods will not discover the same diversity, as the molecular methods recovered fewer annotated species than obtained by morphological analysis, although more OTUs were generated by metabarcoding than by barcoding. Despite reports of the high accuracy of molecular methods (Cowart et al., 2015) and their ability to recover a higher diversity, as demonstrated in the metabarcoding of diatoms (Keck et al., 2018; Rimet, Vasselon, A.‐Keszte, & Bouchez, 2018; Zimmermann, Glöckner, Jahn, Enke, & Gemeinholzer, 2014), in many studies a larger number of species or taxa was identified with traditional microscopy. Examples of the superiority of microscopy in species identification include a study of estuarine plankton, in which 56 taxa were revealed by microscopy but only 37 by metabarcoding (Abad et al., 2016); a study of copepods, in which 54 species were identified morphologically versus. 40 species by metabarcoding (Stefanni et al., 2018); a study of zooplankton, in which 62 species were recovered by morphological analysis and 41–56 species by molecular methods (Harvey et al., 2017); and a deep sea study, in which 20–33 of 35 species were recovered using microscopy (Dell'Anno et al., 2015). Our results fall within this range, as we obtained a comparable diversity based on single‐specimen barcoding for one genetic marker (28S rDNA: 20 OTUs) and morphology (23 species), but a lower diversity using the other marker (18S rDNA: 12 OTUs). Metabarcoding consistently resulted in higher OTU numbers, but the numbers were again higher when 28S rDNA rather than 18S rDNA was used. The 18S gene is generally less variable than the 28S gene such that fewer differences might have accumulated within the barcode region, resulting in a lower resolution and fewer OTUs (Prosser et al., 2013). Several OTUs identified by barcoding that could not be identified by RDP classification could nonetheless be conclusively placed in phylogenetic trees, thus allowing their taxonomic assessment. Despite the high OTU numbers recovered by NGS (28S: 48 OTUs/17 ASVs, 18S: 31 OTUS/6 ASVs), a large proportion could not be classified beyond the genus level. However, the phylogenetic trees showed that all OTUs were reasonably placed, for example, close to the expected species or to OTUs found by single‐specimen barcoding (Figures A1 and A2).

As the true species richness could not be determined using barcoding and metabarcoding approaches, the validity of the comparison with the morphological sample was limited. A more reliable comparison would have been possible if the specimens had been identified prior to Sanger sequencing or metabarcoding, as was the case in other studies (Leasi et al., 2018; Macheriotou et al., 2019). However, our aim was to directly compare the three methods as they are commonly applied. Thus, we could not expect that the studied communities would be identical, but likely very similar and that the more abundant species would be detected by all three methods. While this was indeed the case, taxonomic assignment problems and a lower OTU number obtained with Sanger sequencing limited the comparisons.

Molecular methods are highly sensitive and may eventually complement traditional taxonomy approaches, but our study and those cited above demonstrate the obvious potential of molecular‐based methods to miss species. In our study, the 18S and 28S gene amplifications of the single‐specimen approach resulted in an average sequencing success of 68.6% and 68.8%, respectively, and a combined average of 76%. This rate seems to be typical of single‐specimen barcoding, as the success rate obtained with the COI sequence in an arthropod study was 65.5% (Gibson et al., 2014), and that obtained with 28S and 18S markers in a nematodes study 67% and 98%, respectively (Bhadury et al., 2006; Pereira, Fonseca, Mundo‐Ocampo, Guilherme, & Rocha‐Olivares, 2010). The variability in these rates can be attributed to species that lack an adequate primer‐binding site due to mutations in the primer‐binding regions, as often described for the COI genes (Piñol, Mir, Gomez‐Polo, & Agustí, 2015; Schloss, Gevers, & Westcott, 2011; Suzuki & Giovannoni, 1996). A further reason for the varying efficiencies is the insufficient amounts of DNA often obtained from very small specimens, for example, juveniles or small species in general. Moreover, in many cases, organisms that in the single‐specimen approach could not be amplified with one marker and could also not be amplified with the other marker, perhaps due to poor DNA quality.

Besides the differences in species and OTU/ASV number obtained with barcoding and metabarcoding, taxonomic assignment based on these molecular methods failed in several cases and only three species were identified by all three methods (Figure 2). The most obvious failure was that the OTU most frequently obtained by 28S rDNA barcoding and metabarcoding could not be classified. By contrast, specimens identified by their 28S gene sequence as Sanger_28S_1 were always identified as *Theristus* sp. by the 18S gene sequence. While the correct identification was probably *Theristus agilis*, this species is missing in the databases for both gene fragment sequences (Table 1). However, phylogenetic trees constructed from the two markers placed Sanger_28S_1 and *Theristus* sp. from this study close to the *Theristus* sequences deposited for the markers (Figures A1 and A2) with proportions of both OTUs being similar to that of the morphologically inspected sample identified as *Theristus agilis*. Moreover, for both the 28S and the 18S rDNA gene fragments, some OTUs could only be traced back to the (expected) species based on the morphological data, because of the placement in the phylogenetic background, or by using a second primer pair. Specimens identified as *S. pellucidus* using the 28S gene region were also identified as Sanger_18S_1, and Sanger_28S_7 was identified as *Cylindrolaimus* sp*.* using the 18S gene fragment. As the latter species is completely missing in the 28S rDNA database, the use of a second marker was necessary for its identification. As demonstrated here, an important cause of poor taxonomic assignments using molecular methods is incomplete databases. Consequently, species represented by several reference sequences were more likely to be successfully identified than species lacking accurate species inventories based on a single marker (Table 1). The OTU assignments for the 28S gene fragment resulted in reliable annotations for the OTUs that could be classified as distinct nematode species, which showed that this marker is generally suited for nematode species identification. However, missing sequences in the database prevented many other species‐level assignments (7 of 20 Sanger OTUs classified at the species level). For metabarcoding, 48.3% of the 28S rDNA OTUs but 80.9% of the 18S rDNA OTUs were resolved to the species level.

The performance of the metabarcoding analysis regarding species proportions was to some extent similar to that of the Sanger sequencing approach. The correct depiction of abundance is a known problem for NGS‐based approaches, due to PCR bias and other limitations (Bucklin, Lindeque, Rodriguez‐Ezpeleta, Albaina, & Lehtiniemi, 2016; Elbrecht & Leese, 2015; Vivien, Lejzerowicz, & Pawlowski, 2016). In our study, OTU clustering resulted in higher OTU numbers than obtained with the dada2 pipeline, although this was an unexpected result (as OTUs are basically ASVs clustered at a predefined cutoff). Recently, accurate results with the ASV‐approach using dada2 have been reported (Macheriotou et al., 2019). However, we found that, especially for the 18S gene region, the performance of dada2 was not satisfactory, as it led to the identification of only six ASVs. A similar result was reported in another nematode study, in which a reduced taxonomic assignment was obtained using the dada2 pipeline (Waeyenberge, Sutter, Viaene, & Haegeman, 2019). As the dada2 pipeline uses a more stringent filtering process than the standard OTU‐pipeline, it might omit several sequences maintained in the OTU approach, resulting in a much lower number of reads in the final ASV table construction (OTU: 10,150 reads vs. ASV: 1,015 reads). Despite this potential loss of important information, the filtering out of fewer sequences might result in a high number of OTUs with sequencing artifacts. Therefore, the choice of bioinformatic pipeline should be considered carefully. As we sequenced isolated nematode communities, the more stringent filtering of dada2 was not necessary, but environmental samples (eDNA) might yield different results.

Several initiatives have attempted to curate global biodiversity in public databases for molecular purposes, but these efforts are far from complete (Geiger et al., 2016; Lee et al., 2017). Our study highlights the need to quickly expand molecular databases in order to allow the full use of molecular methods in accurate species assignments. As current databases lack many nematode sequences and a large number of OTUs are of low taxonomic resolution, a taxonomy‐free approach might be preferable until alternative measures become available (Cordier et al., 2018).

In conclusion, our study showed that the results obtained using two genetic markers in barcoding and metabarcoding analyses will improve many of the taxonomic assignments, including those of dominant OTUs, at least to the genus level. This combined approach will compensate for the possible failure of one marker in achieving the correct annotation (as demonstrated in this study by Sanger_18S_1, Sanger_28S_1). Other studies have also emphasized the importance of using more than one marker, if possible, from more than one gene region (Ahmed, Sapp, Prior, Karssen, & Back, 2015; Fontaneto et al., 2015). Furthermore, the obtained results may be more reliable if the gene regions show concordance. Our study highlights the need for fundamental work in species identification and the single barcoding of organisms in order to extend and improve current databases. These efforts will provide insights into taxonomy, body traits, and phylogeny (Fontaneto et al., 2015; Janzen et al., 2005; Pires & Marinoni, 2010). Metabarcoding studies will profit enormously from these efforts, by allowing accurate species‐ or genus‐level identifications. Ecological monitoring, which often relies on exact species assignment and abundance determinations, will also greatly benefit from these efforts.

An integrated approach to species identification based on morphological and molecular analyses will yield a dataset with even greater reliability than based on only one method. Additionally, future methods, such as whole‐genome sequencing and other PCR‐free approaches (Junqueira et al., 2016; Orgiazzi, Dunbar, Panagos, Groot, & Lemanceau, 2015), will eliminate or at least minimize many of the current drawbacks of current molecular approaches, by obviating the need for primers. However, these methods require a high sequencing depth and a much larger number of whole genomes for nematodes in existing databases (Pompanon & Samadi, 2015).

## AUTHOR'S CONTRIBUTIONS

J.S. performed the sampling, laboratory work for the barcoding approach, and wrote the manuscript. W.T. designed the experimental setup and identified nematode species. N.K. conducted the bioinformatic analysis in the metabarcoding approach. All authors contributed in discussions of the study and drafting of the manuscript. None of the authors have any conflict of interest to declare.

## Data Availability

Barcoding sequences of the single specimens were deposited in GenBank under the accession numbers MK379606–MK379948 and MK382985–MK383328. Raw reads of metabarcoding were deposited under PRJNA513975.
