# Supplementary material for: Comparison of morphological, DNA barcoding, and metabarcoding characterizations of freshwater nematode communities
Source: Ecol Evol. 2020 Feb 15;10(6):2885–99. doi: 10.1002/ece3.6104 (PMC7083658; doi:10.1002/ece3.6104)
Supplement: Supplementary file 1 [file ECE3-10-2885-s001.docx]

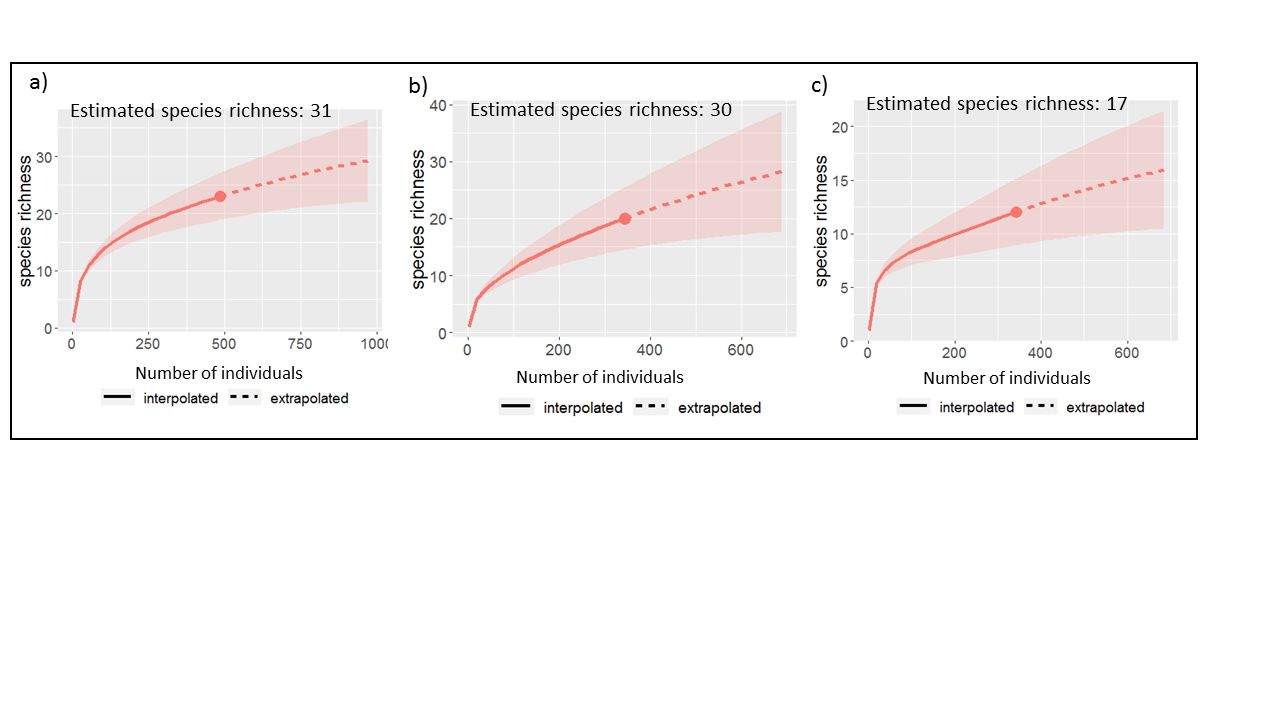


Supplementary Material, Figure S1: Rarefaction curve based on morphologically identified specimens (485) and specimens identified by 28S and 18S rDNA barcoding. The species number for the observed number of individuals and the expected species number if more specimens would be subsequently screened are reported. The estimated species richness based on jackknife estimations is shown for each dataset.
