# Supplementary material for: Comparison of morphological, DNA barcoding, and metabarcoding characterizations of freshwater nematode communities
Source: Ecol Evol. 2020 Feb 15;10(6):2885–99. doi: 10.1002/ece3.6104 (PMC7083658; doi:10.1002/ece3.6104)
Supplement: Supplementary file 2 [file ECE3-10-2885-s002.docx]

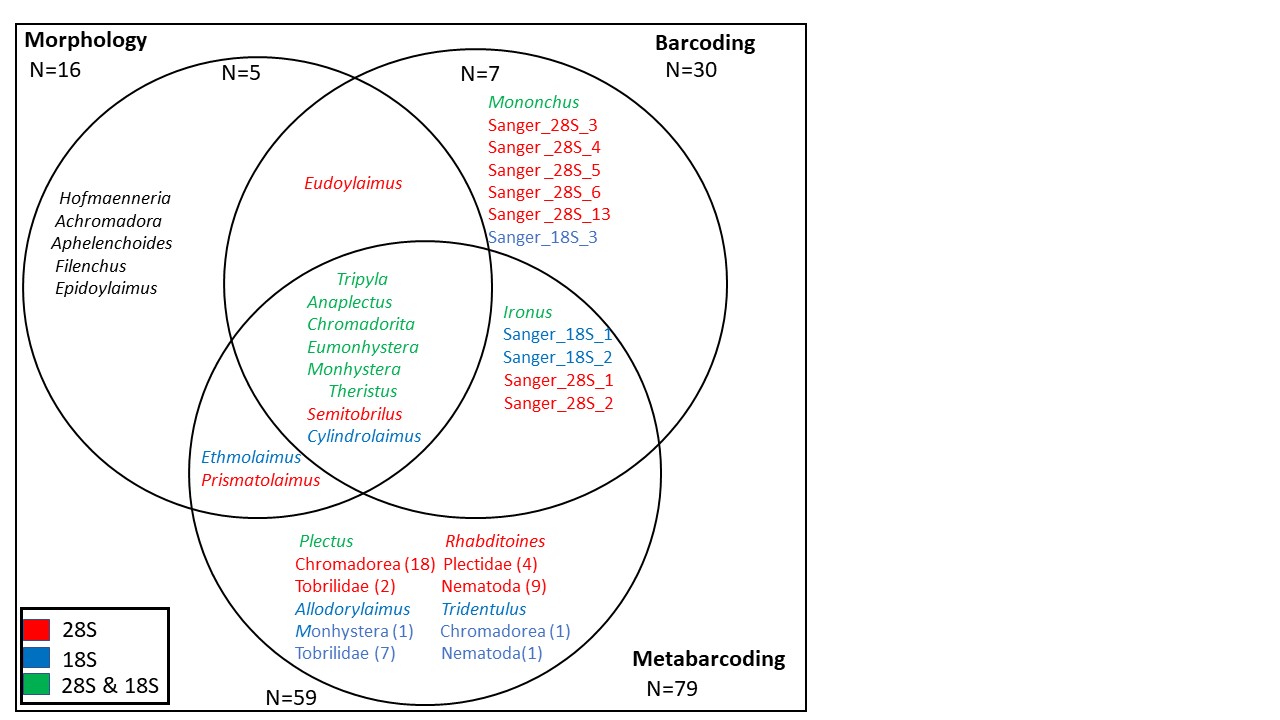


Supplementary Material, Figure S2: Venn Diagram based on the genus level for Morphology, Barcoding and Metabarcoding. The genetic markers are color encoded
