# Supplementary material for: Comparison of morphological, DNA barcoding, and metabarcoding characterizations of freshwater nematode communities
Source: Ecol Evol. 2020 Feb 15;10(6):2885–99. doi: 10.1002/ece3.6104 (PMC7083658; doi:10.1002/ece3.6104)
Supplement: Supplementary file 3 [file ECE3-10-2885-s003.docx]

Supplementary data Table 1 Results of the microscopic analysis. The species abundance of the five replicates combined is indicated together with the sex or life stage (gfem= gravid female, fem=female, mal= male, juv4= juvenile stage 4 and juv=juvenile stage 1-3) of the analyzed nematode.

| Species | gfem | fem | mal | juv4 | juv | total |
| --- | --- | --- | --- | --- | --- | --- |
|  |  |  |  |  |  |  |
| *Achromadora terricola* | 0 | 2 | 0 | 1 | 3 | 6 |
| *Anaplectus grandepapillatus* | 1 | 22 | 20 | 30 | 17 | 90 |
| *Aphelenchoides* sp. | 0 | 0 | 0 | 0 | 1 | 1 |
| *Chromadorita leuckarti* | 9 | 7 | 16 | 5 | 12 | 49 |
| *Cylindrolaimus melancholicus* | 0 | 2 | 1 | 1 | 0 | 4 |
| *Ethmolaimus pratensis* | 1 | 2 | 0 | 0 | 2 | 5 |
| *Eudorylaimus* cf *agilis* | 0 | 0 | 0 | 0 | 1 | 1 |
| *Eudorylaimus* cf *carteri* | 0 | 0 | 1 | 0 | 0 | 1 |
| *Eumonhystera* cf *barbata* | 0 | 1 | 0 | 0 | 0 | 1 |
| *Eumonhystera dispar* | 0 | 1 | 0 | 3 | 0 | 4 |
| *Eumonhystera longicaudatula* | 1 | 6 | 0 | 0 | 2 | 9 |
| *Eumonyhstera pseudobulbosa* | 0 | 1 | 0 | 0 | 0 | 1 |
| *Eumonhystera vulgaris* | 1 | 5 | 0 | 3 | 2 | 11 |
| *Filenchus* sp. | 0 | 0 | 0 | 0 | 1 | 1 |
| *Hofmaenneria niddensis* | 0 | 3 | 4 | 1 | 0 | 8 |
| *Mermithidae* | 0 | 0 | 1 | 1 | 0 | 2 |
| *Monhystera paludicola* | 0 | 1 | 1 | 0 | 0 | 2 |
| *Monhystera stagnalis* | 0 | 1 | 0 | 0 | 0 | 1 |
| *Prismatolaimus intermedius* | 0 | 1 | 0 | 0 | 0 | 1 |
| *Semitobrilus pellucidus* | 12 | 8 | 0 | 2 | 22 | 44 |
| *Theristus agilis* | 3 | 36 | 53 | 43 | 46 | 181 |
| *Theristus vesentinae* | 0 | 0 | 0 | 3 | 13 | 16 |
| *Tripyla setifera* | 7 | 13 | 8 | 9 | 9 | 46 |
|  |  |  |  |  |  |  |
