# Supplementary material for: Comparison of morphological, DNA barcoding, and metabarcoding characterizations of freshwater nematode communities
Source: Ecol Evol. 2020 Feb 15;10(6):2885–99. doi: 10.1002/ece3.6104 (PMC7083658; doi:10.1002/ece3.6104)
Supplement: Supplementary file 4 [file ECE3-10-2885-s004.docx]

Supplementary Material, Table S2: NCBI accession numbers for reference sequences of the 18S and 28S rDNA gene fragments.

| **Species (18S)** | **Accession nb.** | **Species (28S)** | **Accession nb.** |
| --- | --- | --- | --- |
| *Achromadora ruricola* | AY593641.1 | *Pristionchus entomophagus* | MK541653.1 |
| *Achromadora* sp. | Ay284717.1 | *Paroigolaimella bernensis* | MK541654.1 |
| *Achromadora cf. terricola* | AY593640.1 | *Acrostichus nudicapitatus* | MK541655.1 |
| *Anaplectus grandepaillatus* | AY284697.1 | *Acrostichus* sp. | MK541656.1 |
| *Anaplectus porosus* | AY284696.1 | *Rhomborhabditis regina* | MK541657.1 |
| *Anaplectus porosus* | FJ040453.1 | *Panagrellua redivivus* | MK541658.1 |
| *Anaplectus* sp. | AJ966473.1 | *Pristionchus pacificus* | MK541659.1 |
| *Anaplectus* sp. | KY120127.1 | *Caenorhabditis elegans* | MK541660.1 |
| *Aphelenchoides* sp. | GU337999.1 | *Plectus cf. acuminatus* | MK541662.1 |
| *Aphelenchoides* sp. | GU337994.1 | *Plectus aquatilis* | MK541663.1 |
| *Aphelenchoides* sp. | MH844706.1 | *Poikolaimus regenfussi* | MK541666.1 |
| *Aphelenchoides* sp. | GU337996.1 | *Poikolaimus oxycercus* | MK541667.1 |
| *Aphelenchoides* sp. | MH844706.1 | *Diploscpater coronatus* | MK541668.1 |
| *Chormadorita leuckarti* | MF409780.1 | *Acrobeloides tricornis* | MK541665.1 |
| *Chromadorita leuckarti* | FJ969119.1 | *Acrobeloides nanus* | MK541664.1 |
| *Chromadorita leuckarti* | KJ636214.1 | *Allodorylaimus* sp. | KY703629.1 |
| *Chromadorita leuckarti* | KJ636254.1 | *Tobrilus medius* | MF125653.1 |
| *Cylindrolaimus communis* | AY593939.1 | *Tobrilus medius* | MF125659.1 |
| *Cylindrolaimus* sp. | FJ969121.1 | *Plectus* sp. | MF125565.1 |
| *Cylindrolaimus* sp. | AF202149.1 | *Plectus* sp. | KY750806.1 |
| *Ethmolaimus pratensis* | AY593942.1.1 | *Plectus* sp. | MF325283.1 |
| *Ethmolaimus pratensis* | FJ040475.1 | *Plectus velox* | MK541677.1 |
| *Ethmolaimus pratensis* | MG669774.1 | *Plectus opisthocirculus* | KU759362.1 |
| *Eudorylaimus carteri* | AJ966484.1 | *Chiloplectus andrassyi* | KU759360.1 |
| *Eudorylaimus carteri* | MF325111.1 | *Plectus cirratus* | KU759359.1 |
| *Eudorylaimus coniceps* | LC457645.1 | *Plectus aquatilis* | MF125549.1 |
| *Eudorylaimus* sp. | AY284800.1 | *Chromadorita leuckarti* | MF125373.1 |
| *Eumonhystera filiformis* | AY593937.1 | *Triypla setifera* | MF325356.1 |
| *Eumonhystera filiformis* | KJ636219.1 | *Tripyla* sp. | KU921601.1 |
| *Eumonhystera filiformis* | KJ636238.1 | *Tripyla* sp. | MG994928.1 |
| *Eumonhystera filiformis* | KJ636239.1 | *Tripyla setifera* | MF325352.1 |
| *Eumonhystera filiformis* cf. | KJ636240.1 | *Tripyla setifera Gr1122 28S* | MF325352.1 |
| *Eumonhystera hungarica* cf*.* | KJ636237.1 | *Tripyla setifera* | MF125684.1 |
| *Eumonhystera longicaudatula* cf *.* | KJ636252.1 | *Tripyla glomerans* | MF125680.1 |
| *Plectus aquatilis* | MK543230.1 | *Tripyla glomerans* | MF125677.1 |
| *Plectus aquatilis* | GQ892827.1 | *Tobrilus longus* | MF125640.1 |
| *Plectus* sp. | U61761.1 | *Tobrilus longus* | MF125637.1 |
| *Plectus* sp. | MF409867.1 | *Tobrilus longus* | MF125645.1 |
| *Plectus* sp. | JX678607.1 | *Tobrilus pellucidus* | MF125664.1 |
| *Tobrilus gracilis* | KF144671.1 | *Tobrilus pellucidus* | MF125669.1 |
| *Tobrilus gracilis* | AJ966506.1 | *Tobrilus pellucidus* | MF125667.1 |
| *Tobrilus* sp. | KJ636244.1 | *Tobrilus pellucidus* | MF125662.1 |
| *Tobrilus* sp. | GQ503073.1 | *Theristus* sp. | KY792386.1 |
| *Tobrilidae* sp. | KJ636229.1 | *Theristus* sp. | KY792206.1 |
| *Acrobeloides nanus* | KX669638.1 | *Semitobrilus pellucidus* | MF325349.1 |
| *Acrobeloides nanus* | DQ102707.1 | *Prismatolaimus dolichurus* | MF325339.1 |
| *Tridentulus* sp. | AJ966507.1 | *Prismatolaimus dolichurus* | MF325336.1 |
| *Allodorylaimus* sp. | KY942068.1 | *Prismatolaimus* sp. | AB477072.1 |
| *Allodorylaimus* sp. | AJ966472.1 | *Neotobrilus longus* | KF144691.1 |
| *Dichromadora* sp. | FJ040506.1 | *Neotobrilus longus* | KF144689.1 |
| *Dichromadora* sp. | KJ636253.1 | *Neotobrilus longus* | KF144687.1 |
| *Poikilolaimus regenfussi* | MK543233.1 | *Neotobrilus longus* | MF125532.1 |
| *Acrobeloides tricornus* | MK543232.1 | *Mononchus tunbridgensis* | AY593963.1 |
| *Acrobeloides nanus* | MK543231.1 | *Mononchus truncatus* | MF125531.1 |
| *Plectus cf.acuminatus* | MK543229.1 | *Mononchus truncatus* | MF125530.1 |
| *Plectus velox* | MK543228.1 | *Mononchus truncatus* | AY593064.1 |
| *Caenorhabditis elegans* | MK543227.1 | *Mononchus* sp. | KY750781.1 |
| *Pristionchus pacificus* | MK543226.1 | *Mononchus maduei* | MF125526.1 |
| *Panagrellus redivivus* | MK543225.1 | *Mononchus aquaticus* | MF125525.1 |
| *Rhomborhabditis regina* | MK543224.1 | *Monhystera wangi* | MF125522.1 |
| *Acrostichus* sp. | MK543223.1 | *Monhystera stagnalis* | MF125521.1 |
| *Acrostichus nudicapitatus* | MK543222.1 | *Monhystera stagnalis* | MF125518.1 |
| *Paroigolaimella bernensis* | MK543221.1 | *Monhystera stagnalis* | MF125519.1 |
| *Pristionchus entomorphagus* | MK543220.1 | *Monhystera stagnalis* | MF125516.1 |
| *Eumonhystera simplex cf.* | AY284692.1 | *Monhystera* sp. | MF125511.1 |
| *Eumonhystera* sp.*1* | KJ636251.1 | *Monhystera* sp. | MF125503.1 |
| *Eumonhystera vulgaris cf.* | KJ636250.1 | *Monhystera* sp. | MF125508.1 |
| *Filenchus andrassyi* | KJ869339.1 | *Monhystera paludicola* | MF125497.1 |
| *Filenchus aquilonis* | KJ869412.1 | *Monhystera paludicola* | MF125488.1 |
| *Filenchus chilensis* | KJ869411.1 | *Monhystera paludicola* | MF125498.1 |
| *Filenchus discrepans* | AY284591.1 | *Monhystera paludicola* | MF125499.1 |
| *Filenchus filiformis* | AY284592.1 | *Monhystera paludicola* | MF125489.1 |
| *Filenchus hamuliger* | KX156304.1 | *Ironus tenuicaudatus* | MF125483.1 |
| *Filenchus* sp.*1* | MH844388.1 | *Ironus tenuicaudatus* | MF125485.1 |
| *Filenchus* sp.*1* | JQ814875.1 | *Ironus* sp. | MF325228.1 |
| *Filenchus* sp.*1* | FJ949564.1.1 | *Ironus longicaudatus* | MF125481.1 |
| *Filenchus* sp.*2* | JQ814876.1 | *Ironus longicaudatus* | MF125482.1 |
| *Filenchus* sp.*2* | FJ949565.1.1 | *Filenchus vulgaris* | KX156337.1 |
| *Filenchus* sp.*3* | KJ869388.1 | *Filenchus* sp.*5* | JQ005016.1 |
| *Filenchus* sp.*3* | JQ814877.1 | *Filenchus* sp.*4* | JQ005014.1 |
| *Filenchus* sp.*4* | KJ869384.1 | *Filenchus* sp.*3* | JQ005013.1 |
| *Filenchus* sp.*4* | JQ814878.1 | *Filenchus* sp.*2* | JQ80050151 |
| *Filenchus* sp.*5* | JQ814879.1 | *Filenchus* sp.*1* | JQ005012.1 |
| *Filenchus thornei* | KJ869336.1 | *Filenchus* sp.*1* | MH842879.1 |
| *Filenchus vulgaris* | KJ869335.1 | *Filenchus discrepans* | KX156321.1 |
| *Ironus dentifurcatus* | AJ966487.1 | *Eumonhystera filiformis* | MF125480.1 |
| *Ironus elegans* | KC133064.1 | *Eumonhystera filiformis* | MF125476.1 |
| *Ironus longicaudatus* | JQ965925.1 | *Eumonhystera filiformis* | MF125478.1 |
| *Ironus longicaudatus* | FJ040495.1 | *Eumonhystera filiformis* | DQ086658.1 |
| *Ironus macramphis* | KJ636218.1 | *Eudorylaimus* sp.*1* | MF325118.1 |
| *Ironus* sp. | FJ040496.1 | *Eudorylaimus* sp.*1* | MF325220.1 |
| *Ironus* sp. *PM* | AY552970.1 | *Eudorylaimus centrocercus* | AY593007.1 |
| *Monhystera paludicola* | FJ9691301 | *Eudorylaimus carteri* | MF325217.1 |
| *Monhystera paludicola cf.* | KJ636258.1 | *Eudorylaimus altherri* | MF325213.1 |
| *Monhystera riemanni* | AY593928.1 | *Ethmolaimus pratensis* | MF125471.1 |
| *Monhystera* sp.*1* | MF409851.1 | *Chromadorita leuckarti* | MF125359.1 |
| *Monhystera* sp.*1* | KJ636233.1 | *Chromadorita leuckarti* | MF125377.1 |
| *Monhystera* sp.*1* | MF409855.1 | *Aphelenchoides* sp.*1* | Mh844703.1 |
| *Monhystera* sp.*2* | MF409860.1 | *Aphelenchoides* sp.*1* | KT003987.1 |
| *Monhystera* sp.*3* | MF409860.1 | *Aphelenchoides* sp. | KU738610.1 |
| *Monhystera stagnalis* | KJ636259.1 | *Aphelenchoides* sp. | KX356825.1 |
| *Monhystera stagnalis cf.* | KJ636246.1 | *Aphelenchoides* sp. | KX356795.1 |
| *Mononchus aquaticus* | AY284764.1 | *Aphanolaimus* sp. | MF125325.1 |
| *Mononchus aquaticus* | AY284765.1 | *Aphanolaimus aquaticus* | MF125324.1 |
| *Mononchus aquaticus* | KJ636383.1 | *Aphanolaimus aquaticus* | MF125323.1 |
| *Mononchus pulcher* | KJ636382.1 | *Anaplectus* sp.*12* | MG994930.1 |
| *Mononchus truncatus* | AB361451.1 | *Anaplectus porosus* | MF622938.1 |
| *Mononchus truncatus* | AY284762.1 | *Anaplectus granulosus* | MF325171.1 |
| *Mononchus truncatus* | KJ636355.1 | *Anaplectus granulosus* | MF325172.1 |
| *Mononchus tunbridgensis* | AY593954.1 |  |  |
| *Neotobrilus longus* | KF144676.1 |  |  |
| *Neotobrilus longus* | KF144677.1 |  |  |
| *Prismatolaimus intermedius cf.* | KJ636352.1 |  |  |
| *Prismatolaimus intermedius cf.* | KJ636367.1 |  |  |
| *Prismatolaimus intermedius* | AY284729.1 |  |  |
| *Prismatolaimus intermedius* | AF036603.1 |  |  |
| *Prismatolaimus dolichurus* | AY593957.1 |  |  |
| *Prismatolaimus dolichurus cf.* | AY284727.1 |  |  |
| *Prismatolaimus dolichurus cf.* | AY284728.1 |  |  |
| *Semitobrilus pellucidus* | KJ636231.1 |  |  |
| *Theristus agilis* | AY284694.1 |  |  |
| *Theristus agilis TherAgi3* | AY284695.1 |  |  |
| *Theristus* sp. | KX944166.1 |  |  |
| *Theristus* sp. *1* | KX944166.1 |  |  |
| *Theristus* sp. | KX944166.1 |  |  |
| *Theristus acer* | KX944166.1 |  |  |
| *Tobrilus pellucidus* | KR265052.1 |  |  |
| *Tripyla glomerans* | MF409922.1 |  |  |
| *Triypla* sp. | KC485341.1 |  |  |
| *Triypla* sp. | GQ503066 |  |  |
| *Triypla* sp. | GQ503064 |  |  |
| *Triypla* sp. | GQ503072 |  |  |
| *Triypla* sp. | GQ503071 |  |  |
| *Triypla* sp. | KU921595.1 |  |  |
| *Triypla* sp. | KU921596.1 |  |  |
| *Triypla* sp. | KF270637.1 |  |  |
| *Dorylaimus stagnalis* | AY284777.1 |  |  |
| *Dorylaimus stagnalis* | AY284776.1 |  |  |
