# Supplementary material for: Comparison of morphological, DNA barcoding, and metabarcoding characterizations of freshwater nematode communities
Source: Ecol Evol. 2020 Feb 15;10(6):2885–99. doi: 10.1002/ece3.6104 (PMC7083658; doi:10.1002/ece3.6104)
Supplement: Supplementary file 5 [file ECE3-10-2885-s005.docx]

Supplementary Table S3: Results of the molecular analyses. For each specimen analyzed the according sequences of the 18S and the 28S gene fragments are listed with the annotation via RDP classifier.

| **Code** | **1274/706** | | **F04/R22** | | | |  | | **Code** | | **1274/706** | **F04/R22** | |
| --- | --- | --- | --- | --- | --- | --- | --- | --- | --- | --- | --- | --- | --- |
| f1 |  | |  | | | |  | | f101 | | Chromadorita leuckarti | Chromadorita leuckarti | |
| f2 | Monhystera_28S_c | | Eumonhystera_18S_a | | | |  | | f102 | |  | Theristus sp. | |
| f3 |  | |  | | | |  | | f103 | | OTU_28S_1 | Theristus sp. | |
| f4 | Tripyla setifera | | Tripyla_18S_a | | | |  | | f104 | | OTU_28S_1 | Theristus sp. | |
| f5 | Anaplectus granulosus | | Anaplectus porosus | | | |  | | f105 | | Anaplectus granulosus | Anaplectus porosus | |
| f6 | OTU_28S_1 | | Theristus sp. | | | |  | | f106 | | OTU_28S_1 | Theristus sp. | |
| f7 | Eumonhystera filiformis | | Eumonhystera_18S_a | | | |  | | f107 | | Tobrilus pellucidus | OTU_18S_1 | |
| f8 | OTU_28S_1 | | Theristus sp. | | | |  | | f108 | | OTU_28S_1 | Theristus sp. | |
| f9 | Anaplectus granulosus | | Anaplectus porosus | | | |  | | f109 | | OTU_28S_1 | Theristus sp. | |
| f10 | OTU_28S_1 | | Theristus sp. | | | |  | | f110 | | Anaplectus granulosus | Anaplectus porosus | |
| f11 | Anaplectus granulosus | | Anaplectus porosus | | | |  | | f111 | | Chromadorita leuckarti | Chromadorita leuckarti | |
| f12 | OTU_28S_1 | | Theristus sp. | | | |  | | f112 | | OTU_28S_1 | Theristus sp. | |
| f13 | OTU_28S_1 | | Theristus sp. | | | |  | | f113 | | OTU_28S_1 | Theristus sp. | |
| f14 | OTU_28S_1 | | Theristus sp. | | | |  | | f114 | | OTU_28S_1 | Theristus sp. | |
| f15 | Anaplectus granulosus | | Anaplectus porosus | | | |  | | f115 | | Chromadorita leuckarti | Chromadorita leuckarti | |
| f16 | OTU_28S_1 | | Theristus sp. | | | |  | | f116 | | Tobrilus pellucidus | OTU_18S_1 | |
| f17 | OTU_28S_1 | | Theristus sp. | | | |  | | f117 | | Chromadorita leuckarti | Chromadorita leuckarti | |
| f18 | OTU_28S_1 | | Theristus sp. | | | |  | | f118 | | OTU_28S_1 | Theristus sp. | |
| f19 | OTU_28S_1 | | Theristus sp. | | | |  | | f119 | | OTU_28S_1 | Theristus sp. | |
| f20 |  | |  | | | |  | | f120 | | OTU_28S_1 | Theristus sp. | |
| f21 | Monhystera_28S_d | | Eumonhystera_18S_a | | | |  | | f121 | | OTU_28S_1 | Theristus sp. | |
| f22 | OTU_28S_1 | | Theristus sp. | | | |  | | f122 | | Tobrilus pellucidus | OTU_18S_1 | |
| f23 |  | |  | | | |  | | f123 | | Tobrilus pellucidus | OTU_18S_1 | |
| f24 |  | |  | | | |  | | f124 | | OTU_28S_1 | Theristus sp. | |
| f25 | Monhystera_28S_a | |  | | | |  | | f125 | | OTU_28S_1 |  | |
| f26 | OTU_28S_1 | | Theristus sp. | | | |  | | f126 | |  |  | |
| f27 | OTU_28S_1 | | Theristus sp. | | | |  | | f127 | | OTU_28S_1 | Theristus sp. | |
| f28 | OTU_28S_1 | | Theristus sp. | | | |  | | f128 | | OTU_28S_1 | Theristus sp. | |
| f29 | Tobrilus pellucidus | | OTU_18S_1 | | | |  | | f129 | | Anaplectus granulosus | Anaplectus porosus | |
| f30 |  | |  | | | |  | | f130 | | OTU_28S_3 | Anaplectus porosus | |
| f31 |  | |  | | | |  | | f131 | | Anaplectus granulosus | Anaplectus porosus | |
| f32 | Anaplectus granulosus | | Anaplectus porosus | | | |  | | f132 | | Chromadorita leuckarti | Chromadorita leuckarti | |
| f33 | OTU_28S_1 | | Theristus sp. | | | |  | | f133 | | OTU_28S_1 | Theristus sp. | |
| f34 | Tobrilus pellucidus | | OTU_18S_1 | | | |  | | f134 | | Anaplectus granulosus | Anaplectus porosus | |
| f35 | Anaplectus granulosus | | Anaplectus porosus | | | |  | | f135 | | Tobrilus pellucidus | OTU_18S_1 | |
| f36 |  | |  | | | |  | | f136 | | Monhystera_28S_e |  | |
| f37 | Chromadorita leuckarti | | Chromadorita leuckarti | | | |  | | f137 | | OTU_28S_1 | Theristus sp. | |
| f38 |  | |  | | | |  | | f138 | |  |  | |
| f39 | OTU_28S_2 | | OTU-18S_2 | | | |  | | f139 | | Chromadorita leuckarti | Chromadorita leuckarti | |
| f40 |  | |  | | | |  | | f140 | | OTU_28S_1 | Theristus sp. | |
| f41 | OTU_28S_6 | | Eumonhystera_18S_a | | | |  | | f141 | |  |  | |
| f42 | Tripyla setifera | | Tripyla_18S_a | | | |  | | f142 | | OTU_28S_1 | Theristus sp. | |
| f43 |  | |  | | | |  | | f143 | | Chromadorita leuckarti | Chromadorita leuckarti | |
| f44 |  | | OTU_18S_1 | | | |  | | f144 | | Anaplectus granulosus | Anaplectus porosus | |
| f45 |  | | Theristus sp. | | | |  | | f145 | | OTU_28S_1 | Theristus sp. | |
| f46 |  | |  | | | |  | | f146 | | Anaplectus granulosus | Anaplectus porosus | |
| f47 | OTU_28S_2 | | OTU_18S_1 | | | |  | | f147 | | Chromadorita leuckarti | Chromadorita leuckarti | |
| f48 | OTU_28S_1 | | Theristus sp. | | | |  | | f148 | | OTU_28S_1 | Theristus sp. | |
| f49 | Anaplectus granulosus | | Anaplectus porosus | | | |  | | f149 | | Anaplectus granulosus | Anaplectus porosus | |
| f50 |  | | Tripyla_18S_a | | | |  | | f150 | | OTU_28S_1 | Theristus sp. | |
| f51 |  | | Theristus sp. | | | |  | | f151 | | OTU_28S_1 | Theristus sp. | |
| f52 | OTU_28S_1 | | Theristus sp. | | | |  | | f152 | |  |  | |
| f53 |  | | Theristus sp. | | | |  | | f153 | | OTU_28S_1 | Theristus sp. | |
| f54 |  | | Theristus sp. | | | |  | | f154 | |  |  | |
| f55 | OTU_28S_1 | | Theristus sp. | | | |  | | f155 | | OTU_28S_1 | Theristus sp. | |
| f56 |  | | Eumonhystera_18S_a | | | |  | | f156 | | Anaplectus granulosus | Anaplectus porosus | |
| f57 | Tobrilus pellucidus | | OTU_18S_1 | | | |  | | f157 | | Anaplectus granulosus | Anaplectus porosus | |
| f58 |  | |  | | | |  | | f158 | | OTU_28S_2 | OTU_18S_1 | |
| f59 | OTU_28S_1 | | Theristus sp. | | | |  | | f159 | | Anaplectus granulosus | Anaplectus porosus | |
| f60 |  | |  | | | |  | | f160 | | OTU_28S_1 | Theristus sp. | |
| f61 | Anaplectus granulosus | | Anaplectus porosus | | | |  | | f161 | |  |  | |
| f62 | OTU_28S_1 | | Theristus sp. | | | |  | | f162 | |  | Anaplectus porosus | |
| f63 |  | |  | | | |  | | f163 | | OTU_28S_1 | Theristus sp. | |
| f64 |  | |  | | | |  | | f164 | | OTU_28S_2 | OTU_18S_1 | |
| f65 | OTU_28S_1 | | Theristus sp. | | | |  | | f165 | |  | Chromadorita leuckarti | |
| f66 | Tripyla setifera | | Tripyla_18S_a | | | |  | | f166 | |  | Chromadorita leuckarti | |
| f67 | Chromadorita leuckarti | | Chromadorita leuckarti | | | |  | | f167 | | Chromadorita leuckarti |  | |
| f68 | Anaplectus granulosus | | Anaplectus porosus | | | |  | | f168 | | Mononchus truncatus | Mononchus truncatus | |
| f69 |  | |  | | | |  | | f169 | | OTU_28S_1 |  | |
| f70 | OTU_28S_1 | | Theristus sp. | | | |  | | f170 | | OTU_28S_1 | Theristus sp. | |
| f71 | Anaplectus granulosus | | Anaplectus porosus | | | |  | | f171 | |  |  | |
| f72 |  | | Theristus sp. | | | |  | | f172 | | OTU_28S_1 | Theristus sp. | |
| f73 | OTU_28S_1 | | Theristus sp. | | | |  | | f173 | |  | Theristus sp. | |
| f74 | OTU_28S_1 | | Theristus sp. | | | |  | | f174 | |  |  | |
| f75 | OTU_28S_1 | | Theristus sp. | | | |  | | f175 | | Anaplectus granulosus | Anaplectus porosus | |
| f76 |  | |  | | | |  | | f176 | | Tripyla setifera | Tripyla_18S_a | |
| f77 | Anaplectus granulosus | | Anaplectus porosus | | | |  | | f177 | | OTU_28S_1 | Theristus sp. | |
| f78 |  | | Theristus sp. | | | |  | | f178 | | OTU_28S_1 | Theristus sp. | |
| f79 | OTU_28S_1 | | Theristus sp. | | | |  | | f179 | |  |  | |
| f80 |  | | Theristus sp. | | | |  | | f180 | | Chromadorita leuckarti | Chromadorita leuckarti | |
| f81 | OTU_28S_1 | | Theristus sp. | | | |  | | f181 | | Anaplectus granulosus | Anaplectus porosus | |
| f82 | OTU_28S_1 | |  | | | |  | | f182 | | Anaplectus granulosus | Anaplectus porosus | |
| f83 | OTU_28S_1 | | Theristus sp. | | | |  | | f183 | | OTU_28S_1 | Theristus sp. | |
| f84 | OTU_28S_1 | | Theristus sp. | | | |  | | f184 | | Chromadorita leuckarti | Chromadorita leuckarti | |
| f85 | OTU_28S_1 | | Theristus sp. | | | |  | | f185 | |  |  | |
| f86 | OTU_28S_7 | | Cylindrolaimus sp. | | | |  | | f186 | | Anaplectus granulosus | Anaplectus porosus | |
| f87 |  | |  | | | |  | | f187 | | OTU_28S_1 | Theristus sp. | |
| f88 | OTU_28S_1 | | Theristus sp. | | | |  | | f188 | |  |  | |
| f89 | Ironus longicaudatus | | Ironus_18S_a. | | | |  | | f189 | | Anaplectus granulosus | Anaplectus porosus | |
| f90 |  | |  | | | |  | | f190 | | Anaplectus granulosus | Anaplectus porosus | |
| f91 |  | |  | | | |  | | f191 | | Monhystera_28S_c | Eumonhystera_18S_a | |
| f92 | OTU_28S_1 | | Theristus sp. | | | |  | | f192 | |  |  | |
| f93 | OTU_28S_1 | | Theristus sp. | | | |  | | f193 | | OTU_28S_1 | Theristus sp. | |
| f94 | OTU_28S_1 | | Theristus sp. | | | |  | | f194 | | OTU_28S_1 | Theristus sp. | |
| f95 |  | |  | | | |  | | f195 | | Anaplectus granulosus | Anaplectus porosus | |
| f96 | OTU_28S_1 | | Theristus sp. | | | |  | | f196 | |  | Tripyla_18S_a | |
| f97 | Tripyla setifera | | Tripyla_18S_a | | | |  | | f197 | | Tobrilus pellucidus | OTU_18S_1 | |
| f98 | OTU_28S_1 | | Theristus sp. | | | |  | | f198 | | Anaplectus granulosus | Anaplectus porosus | |
| f99 | Anaplectus granulosus | | Anaplectus porosus | | | |  | | f199 | | Anaplectus granulosus | Anaplectus porosus | |
| f100 | Monhystera_28S_a | |  | | | |  | | f200 | | OTU_28S_2 | OTU_18S_1 | |
|  | |  | |  |  | | |  | |  | |  |  |
| f201 | |  | |  |  | | | f301 | | Chromadorita leuckarti | | Chromadorita leuckarti |  |
| f202 | | OTU_28S_1 | | Theristus sp. |  | | | f302 | | OTU_28S_1 | | Theristus sp. |  |
| f203 | | OTU_28S_1 | | Theristus sp. |  | | | f303 | |  | |  |  |
| f204 | | Anaplectus granulosus | | Anaplectus porosus |  | | | f304 | |  | |  |  |
| f205 | |  | |  |  | | | f305 | | OTU_28S_1 | | Theristus sp. |  |
| f206 | | OTU_28S_1 | | Theristus sp. |  | | | f306 | |  | | Chromadorita leuckarti |  |
| f207 | | OTU_28S_1 | | Theristus sp. |  | | | f307 | | OTU_28S_1 | | Theristus sp. |  |
| f208 | |  | | Chromadorita leuckarti |  | | | f308 | |  | | Theristus sp. |  |
| f209 | | OTU_28S_1 | | Theristus sp. |  | | | f309 | | OTU_28S_1 | | Theristus sp. |  |
| f210 | |  | |  |  | | | f310 | |  | |  |  |
| f211 | | Tripyla setifera | | Tripyla_18S_a |  | | | f311 | |  | |  |  |
| f212 | | OTU_28S_1 | | Theristus sp. |  | | | f312 | | OTU_28S_1 | | Theristus sp. |  |
| f213 | | OTU_28S_1 | | Theristus sp. |  | | | f313 | | Anaplectus granulosus | | Anaplectus porosus |  |
| f214 | | Tobrilus pellucidus | | OTU_18S_1 |  | | | f314 | |  | |  |  |
| f215 | | OTU_28S_1 | | Theristus sp. |  | | | f315 | |  | | Theristus sp. |  |
| f216 | | OTU_28S_1 | | Theristus sp. |  | | | f316 | |  | |  |  |
| f217 | | OTU_28S_1 | | Theristus sp. |  | | | f317 | | OTU_28S_1 | |  |  |
| f218 | | Monhystera_28S_b | |  |  | | | f318 | | Chromadorita leuckarti | |  |  |
| f219 | | OTU_28S_1 | | Theristus sp. |  | | | f319 | | Chromadorita leuckarti | | Chromadorita leuckarti |  |
| f220 | | OTU_28S_1 | | Theristus sp. |  | | | f320 | | OTU_28S_1 | |  |  |
| f221 | | OTU_28S_1 | | Theristus sp. |  | | | f321 | |  | |  |  |
| f222 | | OTU_28S_1 | | Theristus sp. |  | | | f322 | |  | |  |  |
| f223 | | Tobrilus pellucidus | | OTU_18S_1 |  | | | f323 | | OTU_28S_1 | | Theristus sp. |  |
| f224 | | Tobrilus pellucidus | | OTU_18S_1 |  | | | f324 | |  | |  |  |
| f225 | |  | |  |  | | | f325 | | Tobrilus pellucidus | | OTU_18S_1 |  |
| f226 | | Tobrilus pellucidus | | OTU_18S_1 |  | | | f326 | | Chromadorita leuckarti | | Chromadorita leuckarti |  |
| f227 | | OTU_28S_1 | | Theristus sp. |  | | | f327 | | Chromadorita leuckarti | | Chromadorita leuckarti |  |
| f228 | | OTU_28S_1 | | Theristus sp. |  | | | f328 | | OTU_28S_1 | |  |  |
| f229 | | Anaplectus granulosus | | Anaplectus porosus |  | | | f329 | | OTU_28S_1 | |  |  |
| f230 | | OTU_28S_1 | | Theristus sp. |  | | | f330 | |  | |  |  |
| f231 | | OTU_28S_1 | | Theristus sp. |  | | | f331 | |  | |  |  |
| f232 | |  | |  |  | | | f332 | |  | |  |  |
| f233 | |  | |  |  | | | f333 | |  | |  |  |
| f234 | |  | | Theristus sp. |  | | | f334 | | Anaplectus granulosus | |  |  |
| f235 | | OTU_28S_1 | | Theristus sp. |  | | | f335 | | Chromadorita leuckarti | | Chromadorita leuckarti |  |
| f236 | | Tobrilus pellucidus | | OTU_18S_1 |  | | | f336 | | OTU_28S_1 | | Theristus sp. |  |
| f237 | | OTU_28S_1 | | Theristus sp. |  | | | f337 | | Tobrilus pellucidus | | OTU_18S_1 |  |
| f238 | | OTU_28S_1 | | Theristus sp. |  | | | f338 | |  | | OTU_18S_1 |  |
| f239 | |  | |  |  | | | f339 | | OTU_28S_1 | | Theristus sp. |  |
| f240 | |  | |  |  | | | f340 | | Tobrilus pellucidus | | OTU_18S_1 |  |
| f241 | | Tobrilus pellucidus | | OTU_18S_1 |  | | | f341 | | OTU_28S_1 | | Theristus sp. |  |
| f242 | | Tobrilus pellucidus | | OTU_18S_1 |  | | | f342 | | Monhystera_28S_a | |  |  |
| f243 | | OTU_28S_1 | | Theristus sp. |  | | | f343 | | Tobrilus pellucidus | | OTU_18S_1 |  |
| f244 | |  | | Theristus sp. |  | | | f344 | | Anaplectus granulosus | |  |  |
| f245 | |  | |  |  | | | f345 | | Anaplectus granulosus | | Anaplectus porosus |  |
| f246 | |  | | OTU_18S_1 |  | | | f346 | | Chromadorita leuckarti | | Chromadorita leuckarti |  |
| f247 | | OTU_28S_4 | | OTU_18S_3 |  | | | f347 | | OTU_28S_1 | | Theristus sp. |  |
| f248 | | Tobrilus pellucidus | | OTU_18S_1 |  | | | f348 | | Tobrilus pellucidus | | OTU_18S_1 |  |
| f249 | | OTU_28S_1 | | Theristus sp. |  | | | f349 | | OTU_28S_1 | | Theristus sp. |  |
| f250 | | OTU_28S_1 | | Theristus sp. |  | | | f350 | | Tobrilus pellucidus | | OTU_18S_1 |  |
| f251 | | OTU_28S_1 | | Theristus sp. |  | | | f351 | |  | |  |  |
| f252 | | Anaplectus granulosus | | Anaplectus porosus |  | | | f352 | | OTU_28S_1 | |  |  |
| f253 | | OTU_28S_1 | | Theristus sp. |  | | | f353 | | Anaplectus granulosus | |  |  |
| f254 | |  | |  |  | | | f354 | | Anaplectus granulosus | | Anaplectus porosus |  |
| f255 | |  | | OTU_18S_1 |  | | | f355 | |  | | Theristus sp. |  |
| f256 | |  | |  |  | | | f356 | | OTU_28S_5 | | Mononchus_18_S_a |  |
| f257 | | Chromadorita leuckarti | | Chromadorita leuckarti |  | | | f357 | | OTU_28S_1 | | Theristus sp. |  |
| f258 | | Ethmolaimus pratensis | | OTU_18S_1 |  | | | f358 | |  | |  |  |
| f259 | | OTU_28S_1 | | Theristus sp. |  | | | f359 | | Anaplectus granulosus | |  |  |
| f260 | | OTU_28S_1 | | Theristus sp. |  | | | f360 | | Tripyla setifera | | Tripyla_18S_a |  |
| f261 | | OTU_28S_1 | | Theristus sp. |  | | | f361 | |  | |  |  |
| f262 | | Tobrilus pellucidus | | OTU_18S_1 |  | | | f362 | | Anaplectus granulosus | | Anaplectus porosus |  |
| f263 | | OTU_28S_1 | | Theristus sp. |  | | | f363 | | OTU_28S_1 | | Theristus sp |  |
| f264 | | OTU_28S_1 | | Theristus sp. |  | | | f364 | | Anaplectus granulosus | |  |  |
| f265 | | OTU_28S_1 | | Theristus sp. |  | | | f365 | | OTU_28S_1 | |  |  |
| f266 | | Anaplectus granulosus | | Anaplectus porosus |  | | | f366 | | Anaplectus granulosus | |  |  |
| f267 | |  | |  |  | | | f367 | |  | |  |  |
| f268 | | Anaplectus granulosus | | Anaplectus porosus |  | | | f368 | | Anaplectus granulosus | | Anaplectus porosus |  |
| f269 | | OTU_28S_1 | | Theristus sp. |  | | | f369 | | Anaplectus granulosus | | Anaplectus porosus |  |
| f270 | | Chromadorita leuckarti | | Chromadorita leuckarti |  | | | f370 | | Tripyla setifera | | Tripyla_18S_a |  |
| f271 | | OTU_28S_1 | | Theristus sp. |  | | | f371 | | Anaplectus granulosus | | Anaplectus porosus |  |
| f272 | | OTU_28S_1 | |  |  | | | f372 | |  | |  |  |
| f273 | | OTU_28S_1 | | Theristus sp. |  | | | f373 | |  | |  |  |
| f274 | | OTU_28S_1 | | Theristus sp. |  | | | f374 | |  | |  |  |
| f275 | | Monhystera_28S_a | | OTU_18S_1 |  | | | f375 | |  | | Theristus sp. |  |
| f276 | | Tobrilus pellucidus | | OTU_18S_1 |  | | | f376 | | OTU_28S_1 | |  |  |
| f277 | |  | |  |  | | | f377 | |  | |  |  |
| f278 | | Anaplectus granulosus | | Anaplectus porosus |  | | | f378 | |  | |  |  |
| f279 | | Tobrilus pellucidus | | OTU_18S_1 |  | | | f379 | | OTU_28S_1 | | Theristus sp. |  |
| f280 | | Tobrilus pellucidus | | OTU_18S_1 |  | | | f380 | |  | |  |  |
| f281 | | Chromadorita leuckarti | | Chromadorita leuckarti |  | | | f381 | |  | | Chromadorita leuckarti |  |
| f282 | | Tobrilus pellucidus | | OTU_18S_1 |  | | | f382 | | Anaplectus granulosus | | Anaplectus porosus |  |
| f283 | | Tobrilus pellucidus | | OTU_18S_1 |  | | | f383 | | OTU_28S_1 | |  |  |
| f284 | |  | |  |  | | | f384 | |  | |  |  |
| f285 | | Tobrilus pellucidus | | OTU_18S_1 |  | | | f385 | | OTU_28S_1 | |  |  |
| f286 | | OTU_28S_1 | | Theristus sp. |  | | | f386 | |  | |  |  |
| f287 | | OTU_28S_1 | | Theristus sp. |  | | | f387 | |  | |  |  |
| f288 | | OTU_28S_1 | | Theristus sp. |  | | | f388 | |  | |  |  |
| f289 | |  | |  |  | | | f389 | | Anaplectus granulosus | |  |  |
| f290 | | OTU_28S_1 | | Theristus sp. |  | | | f390 | |  | |  |  |
| f291 | |  | | OTU_18S_1 |  | | | f391 | | Anaplectus granulosus | | Anaplectus porosus |  |
| f292 | | Tobrilus pellucidus | |  |  | | | f392 | | OTU_28S_1 | | Theristus sp. |  |
| f293 | | OTU_28S_1 | | Theristus sp. |  | | | f393 | | OTU_28S_1 | |  |  |
| f294 | |  | |  |  | | | f394 | |  | |  |  |
| f295 | |  | |  |  | | | f395 | |  | |  |  |
| f296 | | Chromadorita leuckarti | | Chromadorita leuckarti |  | | | f396 | |  | |  |  |
| f297 | |  | |  |  | | | f397 | | Monhystera sp. | |  |  |
| f298 | | OTU_28S_1 | | Theristus sp. |  | | | f398 | |  | |  |  |
| f299 | | OTU_28S_1 | | Theristus sp. |  | | | f399 | |  | |  |  |
| f300 | |  | |  |  | | | f400 | | OTU_28S_1 | |  |  |
|  | |  | |  | |  |  |  |  |  |  |  |  |
| f401 | | OTU_28S_1 | | Theristus sp. | |  |  |  |  |  |  |  |  |
| f402 | |  | |  | |  |  |  |  |  |  |  |  |
| f403 | | OTU_28S_1 | | Theristus sp. | |  |  |  |  |  |  |  |  |
| f404 | |  | |  | |  |  |  |  |  |  |  |  |
| f405 | |  | |  | |  |  |  |  |  |  |  |  |
| f406 | |  | |  | |  |  |  |  |  |  |  |  |
| f407 | |  | |  | |  |  |  |  |  |  |  |  |
| f408 | |  | |  | |  |  |  |  |  |  |  |  |
| f409 | |  | |  | |  |  |  |  |  |  |  |  |
| f410 | |  | |  | |  |  |  |  |  |  |  |  |
| f411 | |  | |  | |  |  |  |  |  |  |  |  |
| f412 | |  | |  | |  |  |  |  |  |  |  |  |
| f413 | |  | |  | |  |  |  |  |  |  |  |  |
| f414 | |  | |  | |  |  |  |  |  |  |  |  |
| f415 | | OTU_28S_1 | | Theristus sp. | |  |  |  |  |  |  |  |  |
| f416 | | Tripyla setifera | |  | |  |  |  |  |  |  |  |  |
| f417 | |  | |  | |  |  |  |  |  |  |  |  |
| f418 | | OTU_28S_1 | | Theristus sp. | |  |  |  |  |  |  |  |  |
| f419 | | Anaplectus granulosus | | Anaplectus porosus | |  |  |  |  |  |  |  |  |
| f420 | |  | |  | |  |  |  |  |  |  |  |  |
| f421 | | Chromadorita leuckarti | | Chromadorita leuckarti | |  |  |  |  |  |  |  |  |
| f422 | | Chromadorita leuckarti | | Chromadorita leuckarti | |  |  |  |  |  |  |  |  |
| f423 | | OTU_28S_1 | | Theristus sp. | |  |  |  |  |  |  |  |  |
| f424 | | Chromadorita leuckarti | | Chromadorita leuckarti | |  |  |  |  |  |  |  |  |
| f425 | | Chromadorita leuckarti | |  | |  |  |  |  |  |  |  |  |
| f426 | | Chromadorita leuckarti | | Chromadorita leuckarti | |  |  |  |  |  |  |  |  |
| f427 | | OTU_28S_1 | | Theristus sp. | |  |  |  |  |  |  |  |  |
| f428 | | OTU_28S_1 | | Theristus sp. | |  |  |  |  |  |  |  |  |
| f429 | |  | |  | |  |  |  |  |  |  |  |  |
| f430 | | Chromadorita leuckarti | | Chromadorita leuckarti | |  |  |  |  |  |  |  |  |
| f431 | | OTU_28S_1 | | Theristus sp. | |  |  |  |  |  |  |  |  |
| f432 | | Anaplectus granulosus | |  | |  |  |  |  |  |  |  |  |
| f433 | |  | |  | |  |  |  |  |  |  |  |  |
| f434 | | OTU_28S_2 | | OTU_18S_1 | |  |  |  |  |  |  |  |  |
| f435 | | Anaplectus granulosus | | Anaplectus porosus | |  |  |  |  |  |  |  |  |
| f436 | |  | |  | |  |  |  |  |  |  |  |  |
| f437 | | OTU_28S_1 | | Theristus sp. | |  |  |  |  |  |  |  |  |
| f438 | | Monhystera_28S_b | |  | |  |  |  |  |  |  |  |  |
| f439 | |  | | Anaplectus porosus | |  |  |  |  |  |  |  |  |
| f440 | | Anaplectus granulosus | | Anaplectus porosus | |  |  |  |  |  |  |  |  |
| f441 | | Anaplectus granulosus | |  | |  |  |  |  |  |  |  |  |
| f442 | | Anaplectus granulosus | | Anaplectus porosus | |  |  |  |  |  |  |  |  |
| f443 | | OTU_28S_1 | | Theristus sp. | |  |  |  |  |  |  |  |  |
| f444 | |  | |  | |  |  |  |  |  |  |  |  |
| f445 | | Tripyla setifera | | Tripyla_18S_a | |  |  |  |  |  |  |  |  |
| f446 | | OTU_28S_1 | | Theristus sp. | |  |  |  |  |  |  |  |  |
| f447 | | OTU_28S_1 | | Theristus sp. | |  |  |  |  |  |  |  |  |
| f448 | |  | |  | |  |  |  |  |  |  |  |  |
| f449 | | Eudoryaimus_28S_a | |  | |  |  |  |  |  |  |  |  |
| f450 | |  | |  | |  |  |  |  |  |  |  |  |
| f451 | |  | |  | |  |  |  |  |  |  |  |  |
| f452 | |  | |  | |  |  |  |  |  |  |  |  |
| f453 | |  | | OTU_18S_1 | |  |  |  |  |  |  |  |  |
| f454 | |  | |  | |  |  |  |  |  |  |  |  |
| f455 | | OTU_28S_1 | | Theristus sp. | |  |  |  |  |  |  |  |  |
| f456 | |  | |  | |  |  |  |  |  |  |  |  |
| f457 | |  | |  | |  |  |  |  |  |  |  |  |
| f458 | |  | |  | |  |  |  |  |  |  |  |  |
| f459 | | OTU_28S_1 | | Theristus sp. | |  |  |  |  |  |  |  |  |
| f460 | |  | | Tripyla_18S_a | |  |  |  |  |  |  |  |  |
| f461 | | Tripyla setifera | | Tripyla_18S_a | |  |  |  |  |  |  |  |  |
| f462 | | Tripyla setifera | |  | |  |  |  |  |  |  |  |  |
| f463 | |  | |  | |  |  |  |  |  |  |  |  |
| f464 | | OTU_28S_1 | |  | |  |  |  |  |  |  |  |  |
| f465 | |  | |  | |  |  |  |  |  |  |  |  |
| f466 | | OTU_28S_1 | | Theristus sp. | |  |  |  |  |  |  |  |  |
| f467 | |  | |  | |  |  |  |  |  |  |  |  |
| f468 | |  | |  | |  |  |  |  |  |  |  |  |
| f469 | | OTU_28S_1 | | Theristus sp. | |  |  |  |  |  |  |  |  |
| f470 | | OTU_28S_1 | | Theristus sp. | |  |  |  |  |  |  |  |  |
| f471 | | OTU_28S_1 | | Theristus sp. | |  |  |  |  |  |  |  |  |
| f472 | | Anaplectus granulosus | | Anaplectus porosus | |  |  |  |  |  |  |  |  |
| f473 | | Chromadorita leuckarti | | Chromadorita leuckarti | |  |  |  |  |  |  |  |  |
| f474 | | OTU_28S_1 | | Theristus sp. | |  |  |  |  |  |  |  |  |
| f475 | | Chromadorita leuckarti | | Chromadorita leuckarti | |  |  |  |  |  |  |  |  |
| f476 | | OTU_28S_1 | | Theristus sp. | |  |  |  |  |  |  |  |  |
| f477 | |  | |  | |  |  |  |  |  |  |  |  |
| f478 | | Chromadorita leuckarti | | Chromadorita leuckarti | |  |  |  |  |  |  |  |  |
| f479 | |  | | Tripyla_18S_a | |  |  |  |  |  |  |  |  |
| f480 | | OTU_28S_1 | | Theristus sp. | |  |  |  |  |  |  |  |  |
| f481 | | Monhystera_28S_d | | Theristus sp. | |  |  |  |  |  |  |  |  |
| f482 | | Tripyla setifera | | Tripyla_18S_a | |  |  |  |  |  |  |  |  |
| f483 | | OTU_28S_1 | | Theristus sp. | |  |  |  |  |  |  |  |  |
| f484 | | OTU_28S_1 | | Theristus sp. | |  |  |  |  |  |  |  |  |
| f485 | | Anaplectus granulosus | | Anaplectus porosus | |  |  |  |  |  |  |  |  |
| f486 | |  | | Tripyla_18S_a | |  |  |  |  |  |  |  |  |
| f487 | | Anaplectus granulosus | | Anaplectus porosus | |  |  |  |  |  |  |  |  |
| f488 | | OTU_28S_1 | | Theristus sp. | |  |  |  |  |  |  |  |  |
| f489 | | OTU_28S_1 | | Theristus sp. | |  |  |  |  |  |  |  |  |
| f490 | |  | | Theristus sp. | |  |  |  |  |  |  |  |  |
| f491 | | OTU_28S_1 | | Theristus sp. | |  |  |  |  |  |  |  |  |
| f492 | | Monhystera_28S_b | | Theristus sp. | |  |  |  |  |  |  |  |  |
| f493 | | OTU_28S_1 | | Anaplectus porosus | |  |  |  |  |  |  |  |  |
| f494 | | Tripyla setifera | | Tripyla_18S_a | |  |  |  |  |  |  |  |  |
| f495 | |  | | Theristus sp. | |  |  |  |  |  |  |  |  |
| f496 | |  | | Chromadorita leuckarti | |  |  |  |  |  |  |  |  |
| f497 | | Tripyla setifera | | Tripyla_18S_a | |  |  |  |  |  |  |  |  |
| f498 | |  | |  | |  |  |  |  |  |  |  |  |
| f499 | |  | | Anaplectus porosus | |  |  |  |  |  |  |  |  |
| f500 | |  | | Tripyla_18S_a | |  |  |  |  |  |  |  |  |
