# Supplementary material for: Comparison of morphological, DNA barcoding, and metabarcoding characterizations of freshwater nematode communities
Source: Ecol Evol. 2020 Feb 15;10(6):2885–99. doi: 10.1002/ece3.6104 (PMC7083658; doi:10.1002/ece3.6104)
Supplement: Supplementary file 6 [file ECE3-10-2885-s006.docx]

Supplementary Table, S4: Overview about bioinformatic workflow. Given is the number of reads for each step and the according read number for the 28S and 18S rDNA marker.

| Reads | 28S | 18S |
| --- | --- | --- |
| reads in total | 12,694 | 12,955 |
| after merging | 9,304 | 12,129 |
| After removal of long/short reads/Homopolymers | 9,304 | 12,129 |
| After removal of reads not fitting to the alignment | 3,768 | 12,123 |
| After uchime | 2,545 | 10,203 |
| After removal of single reads | 2,365 | 10,116 |
