# Supplementary figures and images for: Comparison of morphological, DNA barcoding, and metabarcoding characterizations of freshwater nematode communities
Source: Ecol Evol. 2020 Feb 15;10(6):2885–99. doi: 10.1002/ece3.6104 (PMC7083658; doi:10.1002/ece3.6104)

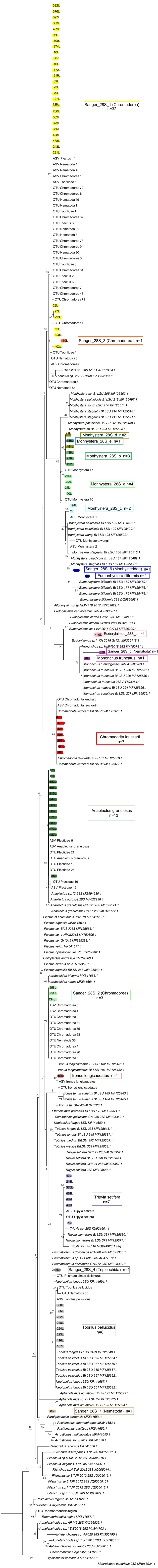

Supplement: Supplementary file 7 [file ECE3-10-2885-s007.pdf]

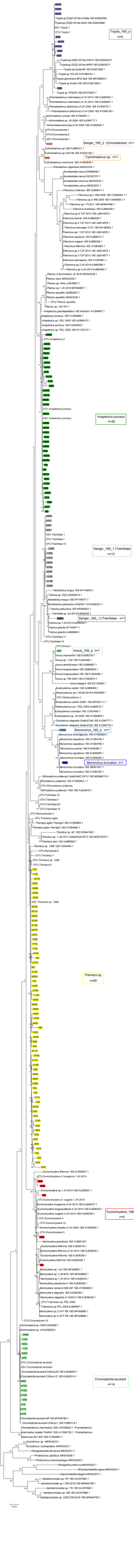

Supplement: Supplementary file 8 [file ECE3-10-2885-s008.pdf]
